# Two lysosomal genes *ATP13A2* and *GBA1* interact to drive neurodegeneration

**DOI:** 10.1186/s13024-025-00923-z

**Published:** 2026-01-30

**Authors:** Mingxue Gu, Jinghan Zhao, Mingxi Deng, Guang Lin, Xueyang Pan, Wenwen Lin, Mengqi Ma, Jinyong Kim, Seul Kee Byeon, Akhilesh Pandey, Lara M. Lange, Chad A. Shaw, Jonggeol Kim, Joanne Trinh, Christine Klein, Oguz Kanca, Joshua M. Shulman, Hugo J. Bellen

**Affiliations:** 1 Department of Molecular and Human Genetics, Baylor College of Medicine, Houston, TX 77030 USA; 2 Department of Neuroscience, Baylor College of Medicine, Houston, TX 77030 USA; 3 Jan and Dan Duncan Neurological Research Institute, Texas Children’s Hospital, Houston, TX 77030 USA; 4 Department of Laboratory Medicine and Pathology, Mayo Clinic, Rochester, MN 55905 USA; 5 Center for Individualized Medicine, Mayo Clinic, Rochester, MN 55905 USA; 6 Manipal Academy of Higher Education, Manipal, Karnataka 576 104 India; 7 Institute of Neurogenetics, University of Luebeck, 23538 Luebeck, Germany; 8 Laboratory of Neurogenetics, National Institute on Aging, Bethesda, MD 20892 USA; 9 Department of Neurology, Baylor College of Medicine, Houston, TX 77030 USA; 10 Center for Alzheimer’s and Neurodegenerative Diseases, Baylor College of Medicine, Houston, TX 77030 USA

**Keywords:** Parkinson’s disease, Lysosomal dysfunction, Elevated glucosylceramide, Glial dysfunction

## Abstract

**Background:**

Parkinson’s disease (PD) is a genetically complex disorder in which combinations of heterozygous risk variants may contribute to pathogenesis. Many PD risk loci encode lysosomal genes, such as *GBA1*, a common and potent risk factor, conferring at least a 5-fold increase. However, the mechanisms of *GBA1* penetrance remain poorly understood.

**Methods:**

Using *Drosophila melanogaster*, we performed a genetic interaction screen of lysosomal storage disorder (LSD) genes to identify dominant modifiers of *Gba1b* (fly homolog of *GBA1*). Age-dependent locomotor assessments, electroretinograms (ERG), transmission electron microscopy (TEM) analyses and quantification of dopaminergic (DA) neurons were used to assess the neurodegenerative phenotypes of double heterozygous animals. By combining immunostaining, lipidomics, metabolomics and pharmacological approaches we showed how partial loss of *anne* (fly homolog of *ATP13A2*) and *Gba1b* drives neurodegeneration. By interrogating genetic data from local and international PD cohorts we identified double heterozygous pathogenic variants in *ATP13A2* and *GBA1* in individuals with PD.

**Results:**

We show that *anne* is expressed in neurons, whereas *Gba1b* is expressed in glia. Flies heterozygous for *anne* exhibit mild neurodegenerative phenotypes, and *Gba1b* strongly enhances this haploinsufficiency. Double heterozygous (*Gba1b*^*T2A*^*/+;anne*^*T2A*^*/+*) flies exhibit a slow and progressive neurodegeneration associated with accumulation and impaired acidification of lysosomes in photoreceptors and other neurons. Obvious morphological defects are first observed in glia at day 15 after eclosion and include vacuolization and neuronal detachment. These defects are accompanied by an elevation of glucosylceramide (GlcCer) and followed by loss of neuronal function and degenerative features by day 30. These phenotypes are neuronal activity-dependent. The neurodegenerative phenotypes are rescued by: ML-SA1, an agonist of the lysosomal TRPML1 channel that has been reported to promote lysosomal membrane trafficking; myriocin, a compound that inhibits GlcCer production; and DFMO, a drug which inhibits polyamine synthesis. Based on surveys of genetic data, we identify multiple PD cases harboring digenic variants in *GBA1* and *ATP13A2*.

**Conclusions:**

Our study reveals that partial loss of *Gba1b* in glia and *anne* in neurons synergistically disrupts lysosomal pH and neuron-glia GlcCer homeostasis, triggering neurodegeneration. Our results provide evidence that *GBA1* penetrance is influenced by additional genetic modifiers, consistent with a putative digenic mechanism for *GBA1*-PD penetrance. These findings highlight lysosomal acidification, sphingolipid clearance, and polyamine regulation as critical intervention points in digenic PD.

**Supplementary Information:**

The online version contains supplementary material available at 10.1186/s13024-025-00923-z.

## Background

Parkinson’s disease (PD), the second most common neurodegenerative disorder, is characterized by a progressive loss of dopaminergic neurons in the substantia nigra and the accumulation of α-synuclein-rich Lewy bodies [1–3]. While environmental factors contribute to its etiology, genetic studies increasingly suggest that PD is also a polygenic disorder and that interactions among multiple genetic risk variants drive neurodegeneration [4]. A growing body of evidence also implicates endo-lysosomal dysfunction in PD susceptibility and pathogenesis, linking rare monogenic LSDs with PD [5–10]. The lysosome is a key degradative and signaling organelle that maintains cellular homeostasis [11, 12]. Its dysfunction disrupts proteostasis and lipid metabolism, creating a permissive environment for α-synuclein aggregation, a hallmark of PD [13, 14]. Genome-wide association studies (GWAS) have consistently identified lysosomal genes as significant PD risk loci, and variants in *GBA1* have emerged as the strongest genetic risk factor that predisposes to PD [15–18].

Biallelic pathogenic *GBA1* variants cause Gaucher disease (GD), an LSD characterized by GlcCer accumulation. In individuals with neuronopathic forms of GD, the variants cause a severe neurodegenerative disease leading to early death [19]. In contrast, heterozygous *GBA1* variants do not cause GD, yet they are the most common genetic risk factors for PD, present in 7–15% of individuals with PD and conferring a fivefold increased risk on average [16, 20, 21]. Over 300 pathogenic *GBA1* variants have been identified, and severe loss-of-function alleles (e.g., p.L444P, p.N370S) are disproportionately associated with earlier PD onset and rapid progression [16, 22–27]. The link between *GBA1* deficiency and PD revolves around sphingolipid dysregulation: *GBA1* encodes glucocerebrosidase (GCase), a lysosomal enzyme that hydrolyzes GlcCer into glucose and ceramide [28]. Reduced GCase activity leads to the elevation of GlcCer and its deacylated form, glucosylsphingosine, which stabilize toxic α-synuclein oligomers, inhibit chaperone-mediated autophagy, and decrease α-synuclein degradation [13, 14].

In the Drosophila brain, *Gba1b* is exclusively expressed in glial cells [29]. We have shown that GlcCer is produced in neurons upon neuronal activity. When the neurons are inactive, GlcCer is internalized through the endo-lysosomal pathway. Fusion of the multivesicular body releases exosomes that contain GlcCer. Upon their uptake in glia, the GlcCer is degraded by the lysosomal Gba1b. Loss of Gba1b results in a progressive lysosomal dysfunction, glial GlcCer accumulation, followed by a neuronal GlcCer accumulation and progressive neurodegeneration, leading to cell death [29]. In contrast, flies heterozygous for *Gba1b* loss-of-function do not exhibit overt neurological defects.

An exome sequencing study of individuals with PD discovered that 56% of the individuals have at least one putative damaging variant in a lysosomal storage disorder gene and that 21% carry multiple pathogenic alleles [30]. This polygenic risk architecture suggests that cumulative lysosomal dysfunction, rather than isolated gene defects, may drive neurodegeneration in PD [30]. While biallelic mutations in these genes cause severe LSDs, heterozygous variants may act as “risk modifiers”. However, which combinations of such variants interact to breach lysosomal thresholds and how remains poorly understood.

Here, we performed a locomotor screen to identify LSD genes that dominantly interact with *GBA1* and discovered that *Gba1b* and *anne*, a conserved homolog of the lysosomal transporter, *ATP13A2*, synergistically drive neurodegeneration. Single heterozygous pathogenic variants in these genes cause no or only minor overt phenotypes, mirroring human heterozygous carriers. However, combined heterozygous loss of *anne* and *Gba1b* results in progressive age-dependent locomotor deficits, functional abnormalities, and cell loss. We show that subtle lysosomal dysfunction in glia and neurons induces progressive neurodegeneration. The accumulation of glucosylceramide (GlcCer) in glia emerges as a pathological driver. Notably, enhancing lysosomal trafficking or blocking GlcCer production mitigates these neurodegenerative phenotypes, providing potential therapeutic insights.

## Methods

### Antibodies, chemicals, and reagents

All reagents and drugs were sourced from EMS, Sigma-Aldrich or Fisher Scientific, unless otherwise specified. A complete list of commercial antibodies, chemicals and assay kits, including vendors and catalog numbers, is provided in Table S5.

### Drosophila stocks and husbandry

Mixed sex of flies (approximately 50% male and 50% female) were used for all experiments. Flies were raised on molasses-based food at 25 °C with constant light unless otherwise noted. The full list of genotypes of the flies used can be found in Table S5.

### Generation of CRISPR mutants

T2A-GAL4 and Kozak-GAL4 mutant flies were generated as previously described [31, 32]. In brief, for the T2A-GAL4 lines, single guide RNAs (sgRNAs) targeting coding introns of LSD genes were designed, along with a homology donor construct containing 200 bp left and right homology arms. The donor construct, which included sgRNA sites for linearization, was synthesized in the pUC57_Kan_gw_OK vector by Genewiz. sgRNAs targeting LSD genes were cloned into the pCFD3 vector. The sgRNAs, homology arms, and a cassette containing the attP-FRT-splice acceptor-T2A-GAL4-poly(A)-3×P3GFP-poly(A)-FRT-attP sequence were subcloned into a homology intermediate vector derived from pM37, generating the final homology donor vector. For Kozak-GAL4 lines, a homology donor intermediate vector encoding sgRNAs targeting regions upstream and downstream of the coding sequence, as well as an sgRNA for linearization, along with 200 bp homology arms, was synthesized in the pUC57_Kan_gw_OK2 vector by Genewiz/Azenta. The Kozak-GAL4-poly(A)-FRT-3×P3GFP-poly(A)-FRT cassette was subcloned into this vector. Homology donor vectors were injected into *nosCas9*-expressing embryos. Adult flies were crossed to *yw* flies, and GFP-positive progeny were selected based on 3×P3-GFP expression in the eyes.

### Cloning and transgenics

Human *ATP13A2* (NM_022089) cDNA was cloned into the destination vector pGW-attB-HA as previously described. Briefly, cDNA in the pDONR221 entry vector was shuttled into the pGW-attB-HA destination vector using Gateway cloning (LR Clonase II, Invitrogen). The *ATP13A2* variant p.R370W was generated via Q5 site-directed mutagenesis (New England Biolabs) in the pDONR221 vector using the following primers:

**Forward: **5′-cccgcagaagagtgtgtgccaccggtgtgtctctgcacagt-3

**Reverse: **5′-actgtgcagagacacaccggtggcacacactcttctgcggg-3

The variant sequence was confirmed by Sanger sequencing following LR recombination. Sequencing primers included gene-specific primers (forward: 5′-aagttgtccatgcgggtgtg-3′; reverse: 5′-atgctgtagatggtgccgag-3′) and standard M13 primers.

For the fly *UAS-anne* line, the clone in the pDONR223 vector was obtained from the Drosophila Genomics Resource Center (Clone ID: 1650874) and transferred to the pGW-attB-HA destination vector using the same Gateway cloning approach. The *anne* p.R491W variant was generated using the following primers:

**Forward: **5′-ttgataaaacagagcatgcctggcatacacttttttgtggcac-3

**Reverse: **5′-gtgccacaaaaaagtgtatgccaggcatgctctgttttatcaa-3

The sequence was verified using gene-specific (5′-ctgtctaaagagcttccaacgcga-3′) and M13 primers.

A genomic rescue construct for *anne*, P{acman;CH321-61L7}, was obtained from BacPac Resources [33]. Transgenic constructs (UAS and genomic rescue) were injected into *y w ΦC31 integrase; VK33* (PBac{y[+]-attP}VK00033) embryos. Final transgenic genotypes included: *y*^*1*^w^*^; PBac{UAS-ATP13A2}VK33/TM3, *y*^*1*^w^*^; PBac{UAS-ATP13A2 p.R370W}VK33/TM3, *y*^*1*^w^*^; PBac{UAS-anne}VK33/TM3, *y*^*1*^w^*^; PBac{UAS-anne p.R491W}VK33/TM3, and *y*^*1*^w^*^; PBac{CH321-61L7}VK00033.

### Genetic screen

We used the Drosophila Integrated Ortholog Prediction Tool (DIOPT) [34] to identify all conserved fly orthologs of 48 human lysosomal storage disorder (LSD) genes [35]. Eight genes (*ASAH1, GM2A, GALC, HYAL1, NEU1, TPP1, CLN5, CLN6*) lack Drosophila homologs and were excluded. For each remaining human gene, we selected high-confidence fly orthologs based on DIOPT rank; when no “high” ortholog existed, we accepted the best forward or reverse match with a DIOPT score ≥10. Nine additional genes (*ARSA, GALNS, GAA, CTSA, GNPTG, CLN8, GRN, KCTD7, LAMP2*) failed to meet these criteria and were likewise excluded. We also incorporated six fly modifiers of neuronal α-synuclein (αSyn) toxicity identified previously [36]. Null allele stocks for each selected fly ortholog were obtained from the Bloomington Drosophila Stock Center (BDSC); the T2A-GAL4 and Kozak-GAL4 mutant alleles were previously generated in our lab. Nine orthologs were excluded due to the unavailability of null allele stocks. Ultimately, we screened 34 Drosophila genes representing 30 human LSD loci.

A total of 34 T2A-GAL4 or Kozak-GAL4 strains were generated. *LSD*^*T2A*^ or *LSD*^*Kozak-GAL4*^ strains were crossed with *w*^*1118*^*;Gba1b*^*KO*^ (*Gba1b*^*STOP*^), and F1 *LSD*^*T2A*^ or *LSD*^*Kozak-GAL4*^*/+;Gba1b*^*STOP*^/+ flies were obtained. Locomotor behavior (see below) was assessed in female adult flies at up to 12 time points between 1 and 30 days post-eclosion. To verify hits, we repeated the screen using an independent *Gba1b*^*T2A-GAL4*^ null allele and confirmed that all phenotypic interactions were allele-independent.

### Robot-assisted locomotor assay

The negative geotaxis climbing assay was conducted using a custom robotic system (SRI International, available at the Automated Behavioral Core of the Duncan Neurological Research Institute), as previously described [37]. Negative geotaxis was induced by tapping custom vials to displace flies to the bottom. Following three taps, high-speed video cameras recorded fly movements at 30 frames per second for 7.5 seconds. For each genotype, 6–8 biological replicates, each consisting of 10–15 female flies, were tested in parallel (biological replicates). Each trial was repeated five times (technical replicates). Replicates were randomly distributed across a 96-vial plate, and all experiments were conducted in a blinded manner. Locomotor performance was quantified based on the average climbing speed in each biological replicate, with individual fly speeds computationally deconvoluted from the video recordings. Robotic assay configuration, video acquisition, and data analysis were performed using the following software packages: Adept Desktop, Video Savant, MATLAB with Image Processing and Statistics Toolkits, RSLogix (Rockwell Automation), and Ultraware (Rockwell Automation). Additionally, custom software was developed for assay control (SRI graphical user interface), analysis [FastPhenoTrack (Vision Processing Software), TrackingServer (Data Management Software), ScoringServer (Behavior Scoring Software)], and visualization [Trackviewer (Visual Tracking Software)].

Locomotor assay data from the robotic *Drosophila* platform were processed by calculating the mean and standard deviation of climbing speed for each genotype across biological replicates, using the *mean()* and *sd()* functions in R. Each experimental genotype was statistically compared to all concurrently tested control genotypes to account for potential temporal and environmental variability. To minimize batch effects, all comparisons were restricted to within-tray analyses. To model age-dependent changes in locomotor performance and assess potential genotype-dependent effects, we employed longitudinal mixed-effects models using the *lme4* package in R [38]. These models included a random intercept to account for genotype-level baseline variation and incorporated cubic B-splines to flexibly capture non-linear time-dependent trends [39]. We tested the differences between all possible pairs of genotypes within each tray by testing the interactions between genotypes and their B-splines using one-way ANOVA (*aov* function in R) with two nested statistical models: (i) a genotype-only model, which tested for mean shifts in climbing speed between genotypes without accounting for changes over time, and (ii) a genotype + time model, which additionally accounted for non-linear time trends. The reported p-values (see Fig. S1) reflect results from both models, with plotted data corresponding to p-values from the genotype + time model. The significance threshold was set at α = 5x10^−4^.

### Climbing assay

Flies were collected at eclosion from a 25 °C constant-dark incubator and aged under either constant-dark or constant-light conditions at 25 °C, with transfers to fresh vials every three days. On the day of behavioral assessment, flies were transferred to an empty vial, tapped to the bottom, and their climbing ability (negative geotaxis) was evaluated. The maximum climbing distance was set at 18 cm, and flies were given 30 seconds to climb. Climbing speeds were subsequently calculated based on the distance climbed within this time frame.

### Electroretinogram (ERG) recording

ERG recordings followed a previously established protocol [40]. Flies were immobilized on glass slides, and two electrodes filled with 0.1 M NaCl were used: one inserted into the thorax and the other placed on the eye. A 1-second pulse of light was applied, and ERG traces were recorded and analyzed using LabChart 8 Reader.

### Transmission electron microscopy (TEM)

Adult fly retinas were prepared for TEM. Fly heads were dissected at 25 °C in fixation buffer containing 2% paraformaldehyde, 2.5% glutaraldehyde, and 0.1 M sodium cacodylate (pH 7.2). Samples were fixed in the same buffer for 48 hours at 4 °C. Following primary fixation, tissues were post-fixed in 1% osmium tetroxide for 40 minutes, then dehydrated through a graded ethanol and propylene oxide series using a Leica EM TP Automated Tissue Processor. Samples were embedded in Embed-812 resin (a mixture of Embed 812, NMA, DDSA, and DMP-30; Electron Microscopy Sciences, Hatfield, PA). Ultrathin sections (50 nm) were cut using a Leica UC7 ultramicrotome and mounted on Formvar-coated copper grids (Ted Pella). Sections were stained with UranyLess and 3% lead citrate (Electron Microscopy Sciences). Images were acquired using a JEOL JEM-1010 transmission electron microscope equipped with an AMT XR-16 mid-mount 16-megapixel CCD camera.

### Immunostaining

For immunostaining of fly retina, heads were dissected in cold 1× phosphate-buffered saline (PBS) and fixed in 3.7% formaldehyde (diluted in PBS) overnight at 4 °C. The following day, the retinas were further dissected in cold PBS and refixed in 3.7% formaldehyde for 30 minutes. Samples were washed in 0.1% Triton X-100 in PBS (PBST), then blocked in 5% natural goat serum (NGS) diluted in 0.1% PBST (5% NGST afterward) for 1 hour at room temperature (RT). After blocking, the samples were incubated for 3 overnights at 4 °C with rabbit anti-GlcCer (1:250; RAS_0010, Glycobiotech). After primary antibody incubation, the samples were washed with 0.1% PBST, followed by incubation with Cy5-conjugated secondary antibody (111–175-144, Jackson ImmunoResearch Labs Inc.) for 1 hour at RT. After incubation with secondary antibodies, the samples were stained by BODIPY 494/504 (D3922, Invitrogen) for 30 minutes at RT. After final washes, samples were mounted in VECTASHIELD (H-1000, Vector Laboratories) and stored at −20 °C before imaging with a Leica SP8 confocal microscope. Retinal confocal images were captured 5 μm below the surface. Fluorescent signals were quantified separately for neuronal and glial regions using ImageJ (Fiji) and Adobe Illustrator.

For immunostaining of fly brains, larval and adult brains were dissected in cold PBS and fixed overnight at 4 °C in 4% paraformaldehyde (diluted in PBS). The following day, brains were permeabilized in 0.2% Triton X-100 in PBS overnight at 4 °C, then blocked in 5% NGST under vacuum at RT for 2 hours. Samples were incubated overnight at 4 °C with primary antibodies: mouse anti-CTSL (1:250; MAB22591, R&D Systems), mouse anti-LBPA (1:100; Z-PLBPA, Echelon Biosciences), rabbit anti-TH (1:200; SAB2701683, Sigma-Aldrich), rat anti-Elav (1:250, Rat-elav-7E8A10, DSHB) and mouse anti-Repo (1:50, Mouse-Repo-8D12, DSHB). After washing in PBST, samples were incubated with Cy3-, or Cy5-conjugated secondary antibodies (Jackson ImmunoResearch Labs Inc.) for 1 hour at RT. Finally, samples were mounted in RapiClear (SunJin Lab Co.) and stored at −20 °C before imaging with a Leica SP8 confocal microscope (Leica) or a Zeiss LSM880 confocal microscope (Carl Zeiss). Images were processed using ImageJ (Fiji) and Adobe Illustrator.

### Western blotting

Fly heads were homogenized in Modified RIPA buffer (50 mM Tris-Cl, 150 mM NaCl, 1% NP-40, 1% sodium deoxycholate, 0.1% SDS, 50 mM NaF, 1 mM Na_3_VO_4_, 10% glycerol and Roche protease inhibitor mix) on ice. Tissue was removed by centrifugation. Isolated lysates were loaded into 4–12% gels, separated by SDS-PAGE, and transferred to PVDF membranes (Millipore). Primary antibodies used in this study were as follows: mouse anti-Actin (1:10,000; ICN691001, ThermoFisher), rabbit anti-Rab5 (1:1,000; ab31261, Abcam), mouse anti-Dynamin (1:1,000; 610245, BD Biosciences), mouse anti-Rab7 (1:500; Rab7, DSHB), rabbit anti-Phospho-Drosophila-S6K (Thr398) (1:1,000; 9209, CST), rabbit anti-S6K (1:1,000; sc-9027, SCBT), rabbit anti-Drosophila Atg8 (1:1,000; gift from S Zhang), rabbit anti-Ubiquitin (P37) (1:1,000; 58395S, CST). Horseradish peroxidase (HRP)-conjugated secondary antibodies (Jackson ImmunoResearch Labs Inc.) were used at 1:5,000.

### Live imaging of LysoTracker

Third instar Drosophila fat bodies or adult brains were dissected in PBS and then stained with 100 nM LysoTracker Red DND-99 (Invitrogen) for 10 min on a shaker. After quickly washing with PBS, samples were mounted in 80% glycerol and were imaged using a Leica SP8 confocal microscope (Leica) or a Zeiss LSM880 confocal microscope (Carl Zeiss).

The number and mean intensity of LysoTracker signals were quantified using Fiji. The images were background-subtracted, and LysoTracker-positive puncta were identified using automatic thresholding. The intensity of each punctum was measured and averaged.

### RNA extraction and quantitative real-time PCR

Total RNA was isolated from 30 adult brains using TRIzol (Invitrogen) and samples were treated with Turbo DNA-free kit (Invitrogen). Reverse transcription was performed using a High-Capacity cDNA Reverse Transcription Kit (Applied Biosystems). RT-PCR was performed using Fast SYBR Green Master Mix (Applied Biosystems) and a QuantStudio 5 Real-Time PCR system (Applied Biosystems). RT-PCR was performed with 3 PCR replicates for each biological sample, 3–4 biological replicates. Primers used for the RT-PCR experiments are listed below.

Primers used:

*Gapdh*: Forward: 5′-CCACTGCCGAGGAGGTCAACTAC-3′; Reverse: 5′-ATGCTCAGGGTGATTGCGTATGC-3′

*vha100-2*: Forward: 5′-ACCCACTTCAAGCGTTATGC-3′; Reverse: 5′-TGAACACCATGTAGCCGAAG-3′

*vhaSFD*: Forward: 5′-GATTTGCCTTTGTGGGAGTC-3′; Reverse: 5′-TTGCACCTGGAAGTTGACAC-3′

*vha16-1*: Forward: 5′-AAGTCTGGTACCGGTATTGC-3′; Reverse: 5′-CCATGACCACAGGAATGATG-3′

*vha68-2*: Forward: 5′-TCATCATCTACGTCGGTTGC-3′; Reverse: 5′-TACGCTTCATGATGGACTCG-3′

*vhaAC45*: Forward: 5′-TCCTGATGGGACTGTTTGTG-3′; Reverse: 5′-TGATGTCCATCATCCAGCAG-3′

*atg8a*: Forward: 5′-ATTCCACCAACATCGGCTAC-3′; Reverse: 5′-GCCATGCCGTAAACATTCTC-3′

*vha100-1*: Forward: 5′-AGCTGCGTTACCTGGAGAAG-3′; Reverse: 5′-TTCTCAAAGGTGGCCTCCAG-3′

*vhaM8.9*: Forward: 5′-GTCCCAAGGCAATCAGTTTC-3′; Reverse: 5′-AAGGGATCGTTGATGGTCAG-3′

### Untargeted lipid analysis via LC-MS/MS

#### Lipid extraction

Lipids were extracted from *w+*, *w+;Gba1b*^*T2A-GAL4*^/+, *w+; anne*^*T2A-GAL4*^/+, *w+;Gba1b*^*T2A-GAL4*^*/+;anne*^*T2A-GAL4*^/+ adult fly heads using a neutral and acidic lipid extraction as described previously [41, 42]. In brief, an internal standard mixture containing deuterated lipids was in equal amount across all samples. Each sample was then mixed with methanol:chloroform to a final volume ratio of 2:1 (v/v) and tip-sonicated. The mixture was briefly vortexed and incubated on ice for 10 minutes to ensure thorough extraction. After centrifugation at 13,800 × g for 2 minutes at 4 °C, 950 μL of the supernatant was collected. To extract remaining lipids, the residual layer was further treated with 750 μL of chloroform:methanol:1 M HCl (40:80:1, v/v/v), vortexed for 1 minute, and incubated at room temperature for 15 minutes. Next, 250 μL of ice-cold chloroform and 450 μL of 0.1 M HCl were added, followed by vortexing for an additional minute. After centrifugation at 6,500 × g for 2 minutes at 4 °C, the lower organic phase was collected and pooled with the first extract. Combined organic extracts were dried under vacuum and stored at − 80 °C until LC-MS analysis.

#### LC-MS/MS for lipidomics

The lipid extracts were analyzed on a Hypersil GOLD Vanquish C_18_ ultrahigh-performance LC (UHPLC) column (150 × 2.1 mm, C_18_ 1.9 μm and 175 Å) using a Fourier transform Orbitrap Fusion Tribrid IQ-X mass spectrometer (Thermo Fisher Scientific) coupled to a Vanquish Horizon UHPLC (Thermo Fisher Scientific) using a data-driven iterative approach as described previously [43]. Untargeted lipidomics were performed as previously described, with modifications. Briefly, a binary gradient at a flow rate of 300 µl min-1 using aqueous phase (water:acetonitrile = 4:6 (vol/vol)) and organic phase (isopropanol:methanol:acetonitrile = 8:1:1 (vol/vol/vol)) with 10 mM ammonium formate and 0.1% formic acid as modifiers was applied to separate the lipids. The organic phase was increased from 20% to 95% over 17 min, maintained at 95% for 3 min and equilibrated to 20% for 5 min for the next injection. The analytical column was maintained at 50 °C. A full-scan mass spectrum (250–1600 m/z in positive ion mode and 200–1600 m/z in negative ion mode) was acquired in the Orbitrap with a resolution of 60,000 at an m/z of 200. MS/MS spectra were obtained at a resolution of 15,000 at an m/z of 200. In positive ion mode, a spray voltage of 3.5 kV and stepped collision energy of 25, 30 and 35% in higher-energy collisional dissociation (HCD) were applied. Collision-induced dissociation (CID) was triggered for ions with detection of m/z 184 in HCD fragmentation. MS^3^ via CID was triggered when neutral loss of fatty acyl chains were detected in the HCD MS/MS for triglyceride characterization. In negative mode, a spray voltage of 2.7 kV and stepped collision energy of 28, 35 and 40% in HCD were used.

#### MS data analysis for lipidomics

The acquired MS/MS spectra were processed using LipidSearch 5 (Thermo Fisher Scientific) to annotate and quantify lipids. Lipids were annotated by comparing precursor ion masses and corresponding MS/MS spectra against the database. Precursor ion mass tolerance was set to 5 ppm, and fragment ion tolerance was set to 8 ppm. All lipids were normalized by peak area of internal standard sharing the identical head group of lipids.

### Metabolon global metabolomics

For each of the twelve genotypes, five pooled biological replicates were prepared, each consisting of 200–250 mixed-sex (1:1 male-to-female ratio) Drosophila heads collected at 7, 15, and 30 days post-eclosion. Heads were isolated by vigorously vortexing snap-frozen flies, followed by separation from bodies using sieves pre-chilled on dry ice. Samples were stored at −80 °C until shipment on dry ice to Metabolon, Inc. for global metabolomic profiling using the HD4 platform. Sample processing, quality control, and initial data analysis were performed by Metabolon. An overview of the HD4 methodology is provided below.

#### HD4 global metabolomics

Sample processing was conducted using the MicroSTAR system (Hamilton Company), during which recovery standards were added. Proteins were precipitated using methanol under vigorous agitation with the GenoGrinder 2000 (Glen Mills). Following extraction, samples were divided into multiple fractions, dried, and reconstituted in appropriate solvents for analysis. Each sample was analyzed under four chromatographic conditions using a Waters ACQUITY UPLC system coupled to a Thermo Scientific Q-Exactive high-resolution/accurate mass spectrometer. This system was equipped with a heated electrospray ionization (HESI-II) source and operated with an Orbitrap mass analyzer at a resolution of 35,000. Metabolite identification was performed using Metabolon’s automated Laboratory Information Management System, which matches ion features in the samples to a proprietary reference library. This library includes annotated metabolites defined by retention time, accurate mass-to-charge ratio (m/z), preferred adducts, in-source fragmentation patterns, and corresponding MS/MS spectra. All identifications were manually curated through visual quality control using Metabolon’s in-house software. For downstream analysis, raw intensity values for each metabolite were normalized to their internal median. Zero values were imputed using the smallest non-zero value detected for that metabolite across the dataset.

#### Data analysis

Log-transformed data from Metabolon were used for PCA and heatmap generation. PCA was performed using the prcomp function in R, and the resulting plot was created with ggplot2 (v3.5.1). The heatmap was generated using the pheatmap package (v1.0.12) with scale = “none”. Pathway annotations were derived from the chemical annotation table provided by Metabolon. Boxplots of individual metabolite levels were created using ggplot2 based on batch-normalized-imputed data.

### Drug administration in fly food

The following chemical inhibitors were added freshly to the regular fly food at the indicated concentration: 10 μM or 100 μM ML-SA1 (29958, Cayman Chemical), 100 μM Myriocin from *Mycelia sterilia* (M1177, Sigma-Aldrich), 10 mM Spermine (S3256, Sigma-Aldrich), 10 mM Spermidine (S2626, Sigma-Aldrich), and 10 mM DFMO (gift from Dr. Stewart and Dr. Casero). The flies were transferred to fresh food with or without the drugs every three days.

### Identification of PD cases with variants in ATP13A2 and GBA1

The institutional review boards at Baylor College of Medicine (BCM) approved both the biospecimen collection and sequencing (H-50254). Subjects were recruited from the Parkinson’s Disease Center and Movement Disorders Clinic during routine clinic visits between 2022 and 2024. All patients with PD were diagnosed by neurologists with subspecialty training in movement disorders. All participants agreed to provide a blood sample for a biorepository along with clinical and demographic data. We confirmed the presence of heterozygous variants in *GBA1* and *ATP13A2* within the BCM proband (along with sibling genotypes) using polymerase chain reaction and Sanger sequencing.

To screen for additional individuals carrying pathogenic or risk variants in both *GBA1* and *ATP13A2*, we further leveraged sequencing data generated by the Global Parkinson’s Genetics Program (GP2; https://gp2.org/) [44]. We used Tier 2 short-read sequencing data from GP2 Release 8 (DOI 10.5281/zenodo.13755496), including clinical exome sequencing (CES) data from 10,454 PD patients and genome sequencing (WGS) data from 5,654 PD patients. Tier 2 data access requires approval and a Data Use Agreement signed by the institution. After removing overlapping samples between both datasets, a total of 15,881 individuals with PD remained for further analyses. GP2’s workflow, as well as sequencing, genotyping, processing, and quality control of genetic data within GP2, have been described before [45–47]. Variants in the genes of interest were extracted using PLINK [48, 49] and annotated with ANNOVAR [50, 51]. We used the Gauchian pipeline (https://github.com/Illumina/Gauchian) [52] for WGS data as a variant caller for *GBA1*. All code generated for this article, and the identifiers for all software programs and packages used, are available on GitHub (link) and were given a persistent identifier via Zenodo.

We also interrogated genome sequencing from the UKBB (UK Biobank) to identify the frequency of *ATP13A2* variants in unaffected *GBA1* carriers. We accessed UKBB data in June 2025 via DNAnexus under application number 33601. To determine PD status, we used Data Field “Source of report of G20 (parkinson’s disease)” (field 131023). For the sequencing data, we used the DRAGEN population level WGS data (field 24310). WGS processing and quality control has been described elsewhere [53, 54]. The variants in the genes of interest were extracted using PLINK and annotated with VEP [55]. In total, we identified 25,869 *GBA1* carrying participants without PD report.

### Quantification and statistical analysis

All datasets were analyzed using Microsoft Excel 365 and GraphPad Prism (10.4.2). Statistical comparisons were performed using two-tailed Student’s t-tests or one-way analysis of variance (ANOVA), as appropriate. Sample sizes for fly experiments are provided in Figure legends, with all crosses performed at least three times. Data are presented as mean ± SEM. Statistical significance thresholds are as follows: ns (not significant), *p* > 0.05; *p < 0.05; **p < 0.01, ***p < 0.001, and ****p < 0.0001.

## Results

### *Gba1b *dominantly interacts with *anne*

In order to test the hypothesis that loss-of-function in other LSD genes may modify *GBA1* penetrance in PD, we screened for dominant interactions of *Gba1b* with other fly orthologues of LSD genes, using a simple climbing assay in aging double heterozygous flies (*Gba1b*^-^*/+;LSD*^-^/+). We screened a total of 34 fly orthologues in combination with one *Gba1b* null allele [56, 57] (Table S1). Alleles for each *LSD* were crossed with *Gba1b*^*STOP*^ (*LSD*^-^*/+; Gba1b*^*STOP*^/+), a severe loss-of-function allele [58].

We measured the negative geotactic response of flies using an automated locomotor behavioral assay [59] and measured climbing speed of adult *LSD*^-^/+ and *LSD*^-^*/+; Gba1b*^*STOP*^/+ flies longitudinally from day 0 to day 30. The single loss of nine genes—*Csp*, *Ect3*, *Sgsh*, *CG5731*, *Npc1a*, *Snmp1*, *Trpml*, *emp*, *and anne*—causes a significant decline in climbing ability with aging compared to controls (Table S1, Fig. S1A-I). Among these, only *anne*, the Drosophila ortholog of human *ATP13A2*, showed a further deterioration in locomotor phenotypes when combined with *Gba1b*, suggestive of a synergistic interaction (Table S1, Fig. S1I). Hence, we focused on the interaction between *anne* and *Gba1b*.

*ATP13A2* (or *PARK9*) encodes a lysosomal ATPase that exports polyamines from the lysosomes and mediates K^+^/H^+^ exchange [60, 61]. Biallelic *ATP13A2* variants cause Kufor-Rakeb syndrome, a juvenile-onset parkinsonism with dementia [57]. *ATP13A2* deficiency elevates α-synuclein levels and increases neuronal susceptibility to oxidative stress [63–65]. The human ATP13A2 and fly Anne proteins share 36% identity and 52% similarity in amino acid composition (DIOPT score: 13/19) and the protein structures are highly conserved as predicted by AlphaFold [66, 67].

### Loss of *anne* causes embryonic lethality

**Fig. 1:**
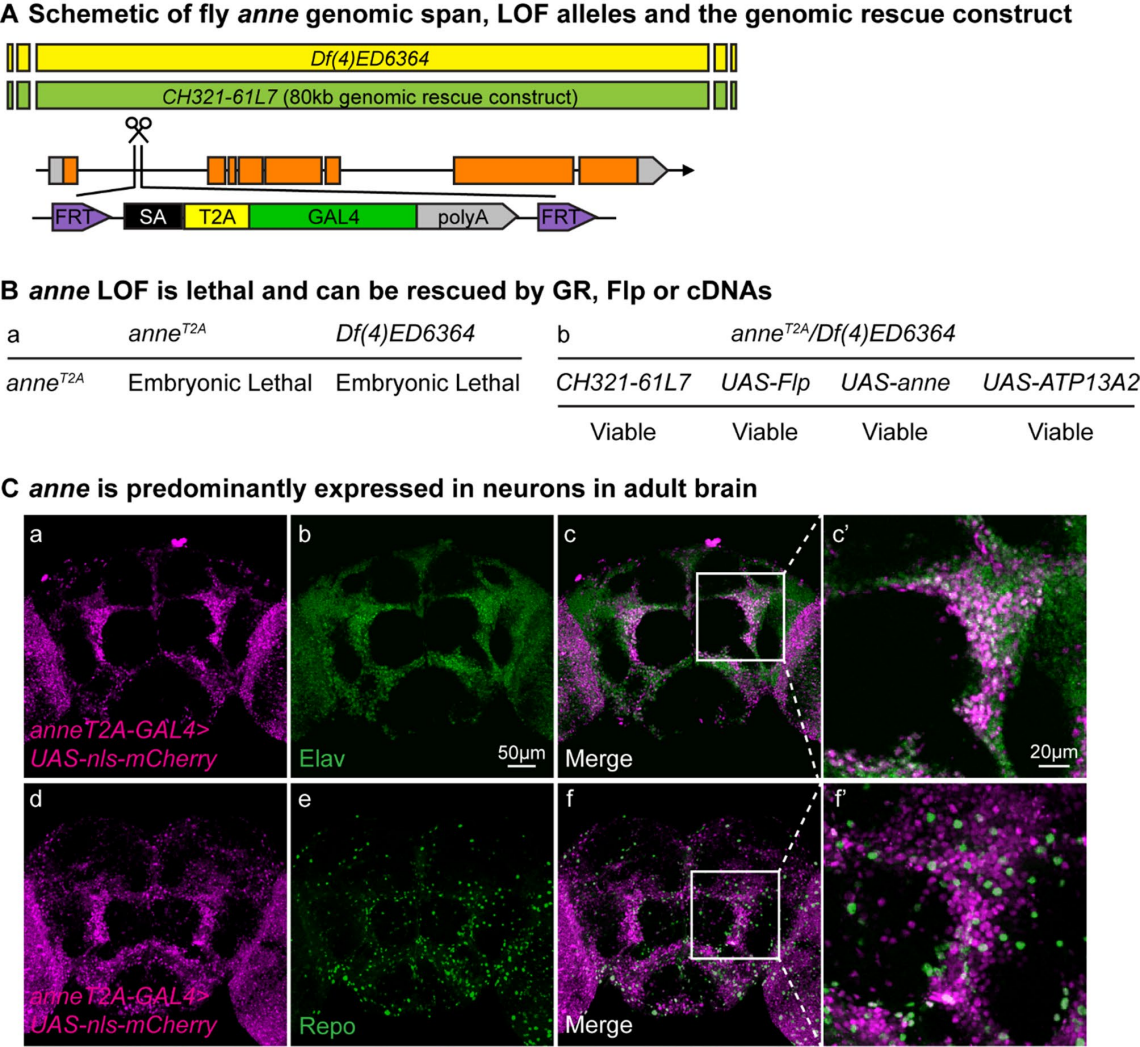
*anne* is predominantly expressed in neurons. (**A**) Genomic structure of *anne* locus and reagents used in this study. (**B**) Summary of the lethality phenotype of flies lack *anne*: (i) *y*^*1*^w^*^;anne^*T2A-GAL4/T2A-GAL4*^, (ii) *y*^*1*^w^*^;anne^*T2A-GAL4*^*/Df*. Lethality can be fully rescued by a genomic fragment (GR, CH321-61L7), Flippase (UAS-Flp), and reference fly and human cDNAs (*n* > 100). (**C**) Expression pattern of *anne* in the adult brain is visualized using *anne*^*T2A-GAL4*^ allele-driven expression of *UAS-mCherry.NLS* (magenta) co-stained with markers for neurons (Elav) or glia (Repo) (green). Single-layer confocal images from the dashed squares indicate that mCherry is co-localized with Elav (c’) but not Repo (f’). Scale bars, 50 μm and 20 μm. *n* > 3

To assess the phenotypes associated with the loss of function of *anne,* we used the *anne*^*T2A-GAL4*^allele (Material and Methods) as well as a CRISPR allele, *anne*^*1-205AA*^ that truncates the protein and only retains 205 of the 1047 amino acids [68] (Fig. S2A). In addition, we used the *Df(4)ED6366* (*Df* for short), which is a chromosomal deletion allele that spans about 120 kb, removing all *anne* coding sequences [69] (Fig. 1A). Biallelic LoF *anne* alleles result in embryonic lethality (Fig. 1B, a, Fig. S2B). Moreover, the following three experiments unambiguously show that the embryonic lethality is indeed due to the T2A-GAL4 insertion. First, the lethality is fully rescued by the introduction of one copy of an 80-kb *P{acman;CH321-61L7}*, a genomic bacterial artificial chromosome (BAC) that contains *anne*. Second, by using a Flippase (UAS-Flp), the FRT sites that flank the T2A-GAL4 cassette can be utilized to excise the mutant cassette and revert the phenotype. Finally, the GAL4 permits the rescue of embryonic lethality of mutant animals to adult flies by driving the *UAS-anne* or *UAS-human ATP13A2* cDNAs (Fig. 1B, b). The latter also shows that the human protein can fully rescue the loss of the fly gene and that their function is evolutionarily conserved.

**Fig. 2:**
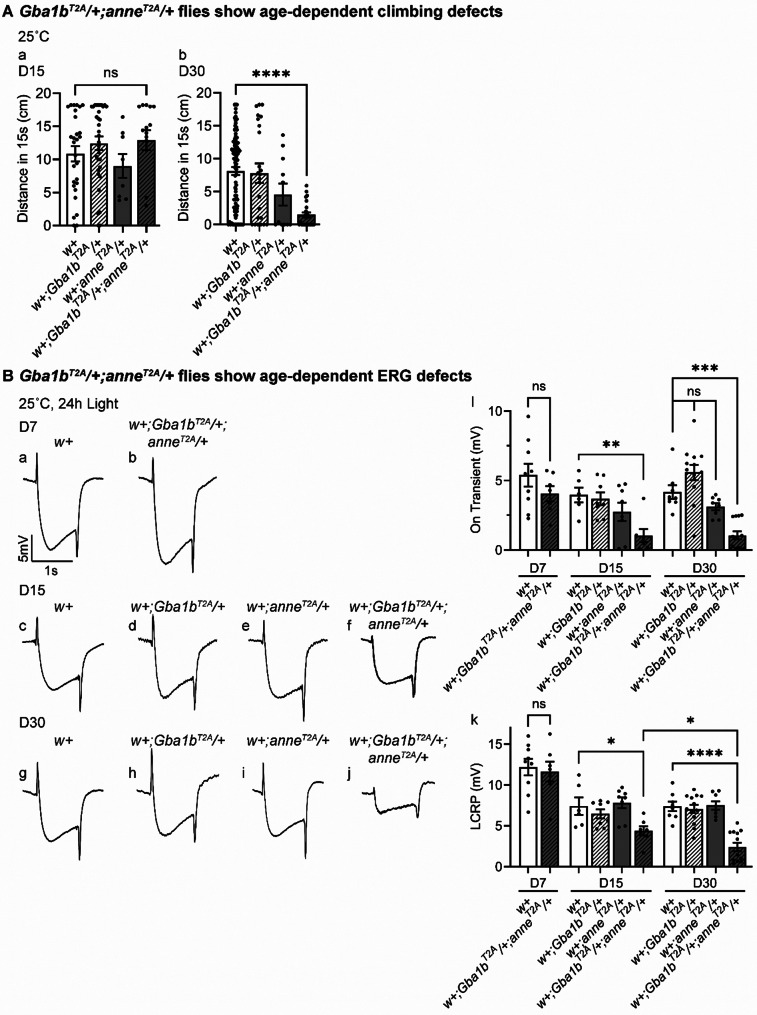
*anne* genetically interacts with *Gba1b* and mediates age-dependent loss of neuronal activity. (**A**) Climbing ability of flies of the indicated genotypes after 15 or 30 days. *w+;Gba1b*^*T2A*^*/+;anne*^*T2A*^*/+* flies have severe climbing defects at D30. Flies were raised at 25 °C (*n* > 20). (**B**) ERG recordings of flies of the indicated genotypes after 7, 15 or 30 days of constant light. *w+;Gba1b*^*T2A*^*/+;anne*^*T2A*^*/+* flies show a reduction of LCRPs and on-transients from D15. The ERG LCRP and on-transient amplitudes are quantified on the right (*n* > 6). Error bars represent SEM; *p < 0.05, **p < 0.01, ***p < 0.001, ****p < 0.0001

**Fig. 3:**
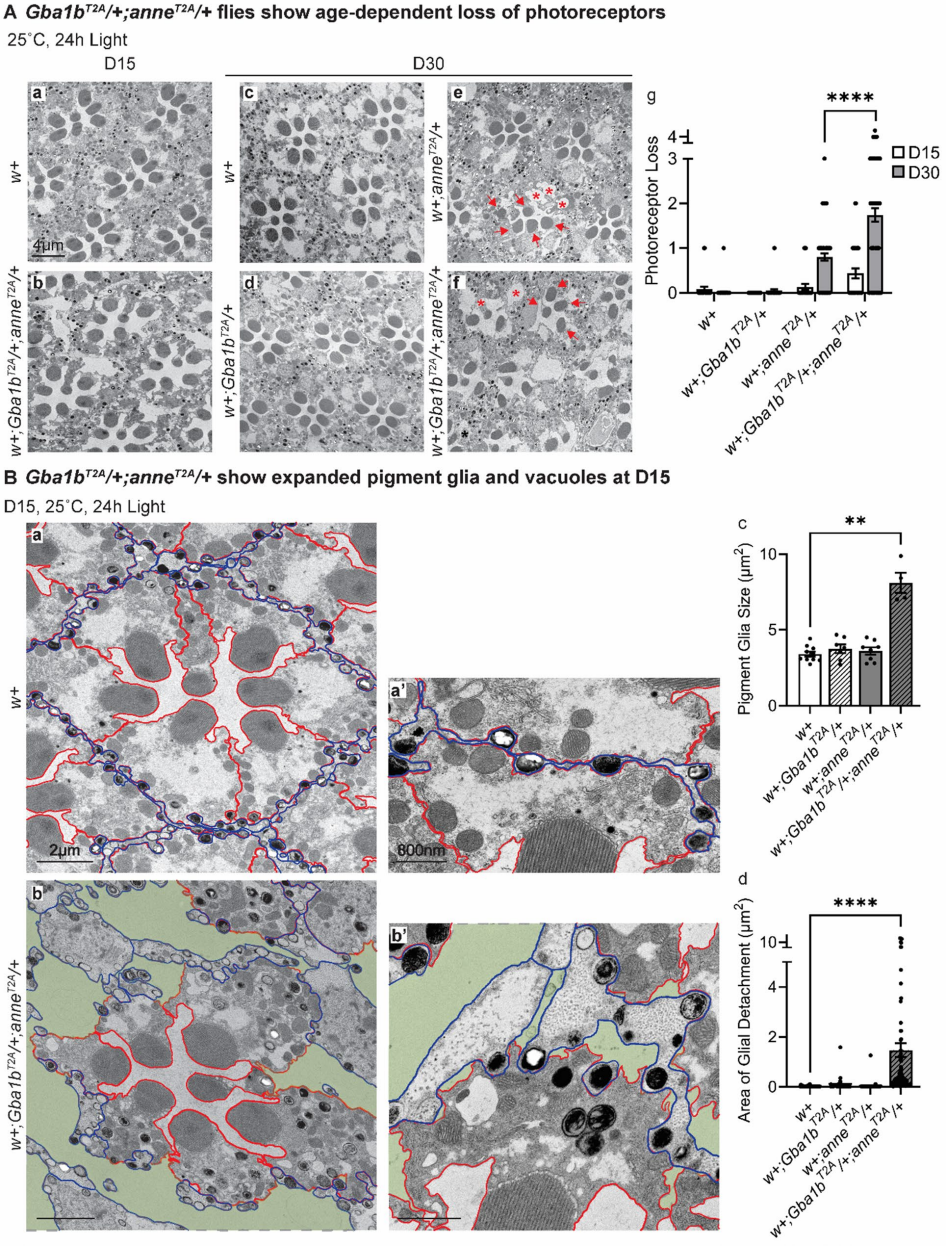
Aged *Gba1b*^*T2A*^*/+;anne*^*T2A*^/+ flies show impaired glial morphology, which precedes neuronal loss. (**A**) TEM images of fly retinas of the indicated genotypes after 15 or 30 days of constant light. Red arrows point to rhabdomeres. Red asterisks point to vacuoles. The overall morphology of the retina is severely affected in *w+;Gba1b*^*T2A*^*/+;anne*^*T2A*^/+ flies, whereas the retinas of *w+* or *w+;Gba1b*^*T2A*^/+ flies do not show obvious defects. *w+;anne*^*T2A*^/+ flies show mild defects. The loss of intact photoreceptors per ommatidium is quantified on the right (*n* > 30). Retinas from 3 animals were quantified. Scale bars, 4 μm. (**B**) Enlarged TEM images of a single ommatidium of the indicated genotypes after 15 days of constant light. Pigment cells are highlighted in blue. Photoreceptors are highlighted in red. Green marks glial detachment, which is commonly seen in the retina of *w+;Gba1b*^*T2A*^*/+;anne*^*T2A*^/+ flies, but rarely observed in *w+* flies. The size of pigment glia and the area of glial detachment per ommatidium are quantified on the right (*n* > 20). Retinas from 3 animals were quantified. Scale bars, 2 μm and 800 nm. Error bars represent SEM; *p < 0.05, **p < 0.01, ***p < 0.001, ****p < 0.0001

### *anne *is expressed in neurons

To determine the expression pattern of *anne* in the central nervous system (CNS), *anne*^*T2A-GAL4*^*/in(4)ci*^*D*^ animals were used to drive *UAS-nls:mCherry*, allowing the identification of nuclei of cells that express *anne*. Co-staining with antibodies against Elav (a neuronal marker) and Repo (a glial marker) revealed that *anne* is primarily expressed in neurons within the ventral nerve cord of third-instar larvae (Fig. S3A). In adult brains, *anne* is expressed in most neurons but only in ~10% of glia of all brain regions (Fig. 1C). The *anne* expression suggests that it is likely expressed in active neurons during development [70]. In summary, *anne* is mostly expressed in neurons in the CNS.

### *anne *and *Gba1b* double heterozygous mutants exhibit progressive neurodegeneration

The climbing screen data suggest that *anne* and *Gba1b* (*Gba1b*^*STOP*^*/+;anne*^*T2A-GAL4*^/+) genetically interact to promote neurodegeneration. To validate this interaction, we assayed other combinations of loss-of-function alleles: *Gba1b*^*T2A-GAL4*^*/+;anne*^*T2A-GAL4*^/+ and *Gba1b*^*STOP*^*/+;anne*^*1-205AA*^/+ flies. Consistent with our previous observation, both genotypes lead to significant climbing impairments when compared to *Canton-S* or *y*^*1*^w* controls (Fig. 2A and Fig. S2C).

**Fig. 4:**
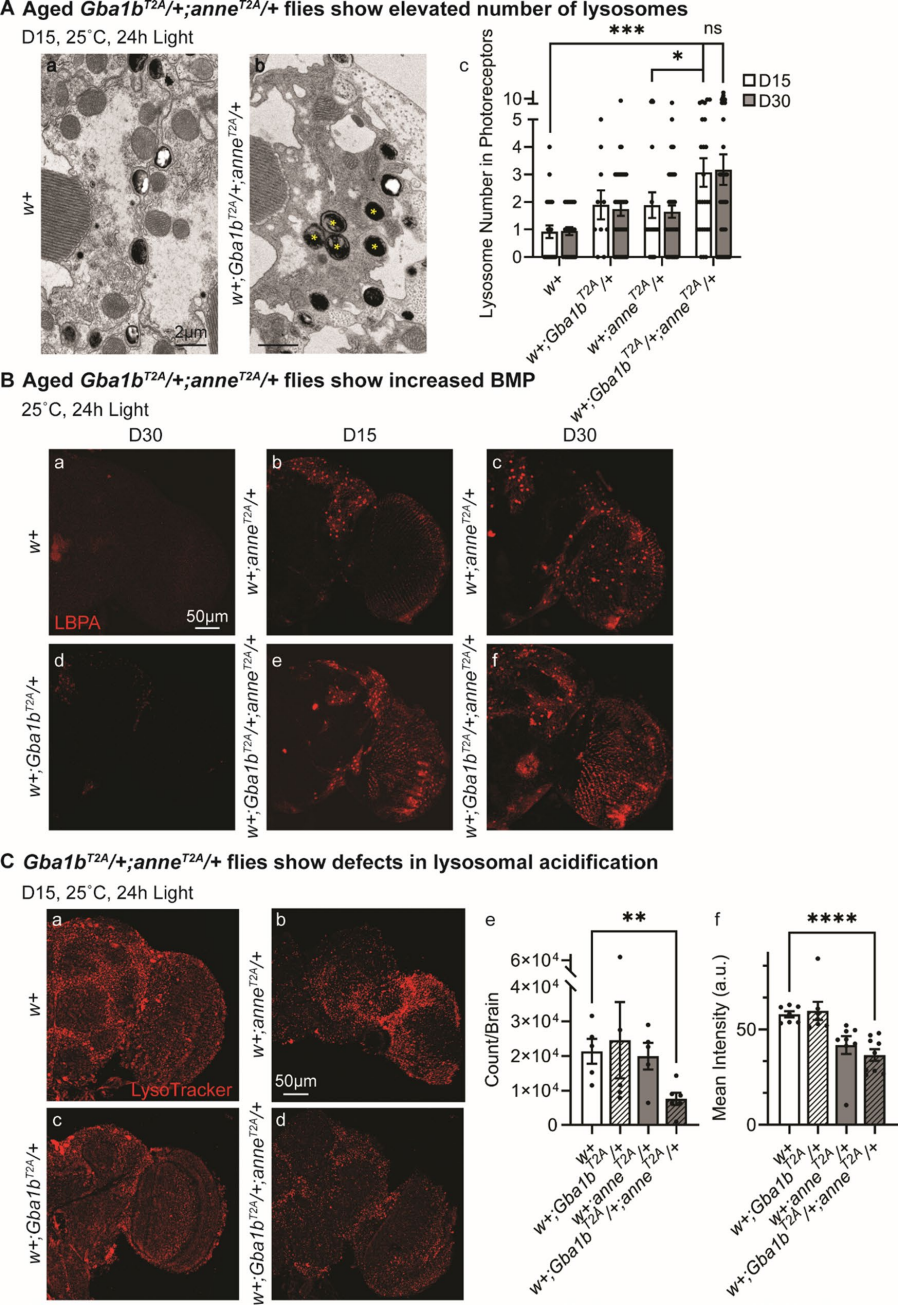
Aged *Gba1b*^*T2A*^*/+;anne*^*T2A*^/+ flies show an elevated number of lysosomes but reduced lysosomal acidification in the neurons. (**A**) Enlarged TEM images of a photoreceptor of the indicated genotypes after 15 days of constant light. Yellow asterisks point to lysosomes in the photoreceptors. *w+;Gba1b*^*T2A*^*/+;anne*^*T2A*^/+ flies exhibit an increased number of lysosomes in photoreceptors at D15 when compared to *w+*, *w+;Gba1b*^*T2A*^/+, or *w+;anne*^*T2A*^/+ flies (*n* > 20). Scale bars, 2 μm. The number of lysosomes in the photoreceptors is quantified on the right. Retinas from 3 animals. (**B**) Immunostaining of LBPA in the brains of indicated genotypes after 15 or 30 days of constant light. *w+;Gba1b*^*T2A*^*/+;anne*^*T2A*^/+ flies have increased fluorescent intensity of BMP at D15 when compared to *w+* or *w+;Gba1b*^*T2A*^/+ flies. *w+;anne*^*T2A*^/+ flies show a mild increase in BMP fluorescent intensity. Scale bars, 50 μm. *n* = 3. (**C**) Live imaging of LysoTracker Red DN-99 in the brains of indicated genotypes after 15 days of constant light. *w+;Gba1b*^*T2A*^*/+;anne*^*T2A*^/+ flies show defective lysosomal acidification. Scale bars, 50 μm. The number and fluorescence intensity of the LysoTracker positive puncta are quantified in e and f. Error bars represent SEM; *p = 0.0184, **p = 0.0062, ****p < 0.0001

To assess the roles of *anne* and *Gba1b* in neurons and glia, we used the Drosophila visual system as a model. Electroretinogram (ERG) recordings allow us to assess photoreceptor function and synaptic transmission. Wang et al. (2022) [29] documented that prolonged light-induced neuronal activity leads to severe ERG defects when *Gba1b* is lost. In addition, loss of *white* exacerbates the phenotypes and loss of *white* in an otherwise wild-type background leads to a progressive glial dysfunction and accumulation of GlcCer in pigment glia. Hence, we assayed all genotypes in a *white+* background (*Canton-S* (w+), abbreviated *w+*) or with the *Gba1b*^*STOP*^ allele that carries a *white+* allele.

To assess if there is a progressive loss of neuronal function, we recorded ERGs in 7-, 15-, or 30-day-old flies exposed to continuous light. Single heterozygous *w+;Gba1b*^*T2A-GAL4*^/+ flies or *w+;anne*^*T2A-GAL4*^/+ flies do not exhibit ERG defects (Fig. 2B, d, e, h, i, l, k). In contrast, double heterozygous *w+;Gba1b*^*T2A-GAL4*^*/+;anne*^*T2A-GAL4*^/+ flies display a moderate reduction in light coincident receptor potential (LCRP) amplitudes and on-transients by day 15 (Fig. 2B, f, l, k). However, by day 30, we observe a severe reduction in LCRP amplitude and both on/off-transients (Fig. 2B, j-l). *y*^*1*^w*;Gba1b^*STOP*^*/+;anne*^*T2A-GAL4*^/+ and *y*^*1*^w*;Gba1b^*STOP*^*/+;anne*^*1-205AA*^/+ flies also show a significant LCRP amplitude decrease compared to *y*^*1*^w*;Gba1b^*STOP*^/+ controls when aged (Fig. S2D). Together, our data show that a partial loss of *anne* in neurons and *Gba1b* in glia synergistically disrupts phototransduction and synaptic transmission.

### Glial morphology is affected prior to neuronal loss in *Gba1b*^-^*/+;anne*^-^*/+ ***flies**

To investigate the ultrastructural defects, we turned to the fly eye. The fly eye comprises ~750 ommatidia, each containing eight photoreceptor neurons surrounded by pigment glia. We performed transmission electron microscopy (TEM) of flies aged for 15 and 30 days. After 15 days of light exposure, the *w+;Gba1b*^*T2A-GAL4*^*/+;anne*^*T2A-GAL4*^/+ mutants display a mild loss of photoreceptors (Fig. 3A, a, b, g) while glial cells are obviously disrupted (Fig. 3B, a-b’, circled in blue). While glia in control flies are closely associated with photoreceptors, glia of the double heterozygous mutants are often swollen (Fig. 3B, a-c) and detached from the neighboring photoreceptors (Fig. 3B, a-b’, highlighted in green; 3B, d). In contrast, little to no obvious morphological defects were observed in photoreceptors at day 15 (Fig. 3B, a-b’), indicating that the primary structural abnormalities originate in glia.

By day 30, TEM revealed neurodegenerative phenotypes in *w+;Gba1b*^*T2A-GAL4/*^*+;anne*^*T2A-GAL4*^/+ mutants, including photoreceptor loss, vacuolization, and glial loss (Fig. 3A, c-g, red arrowheads and asterisks), consistent with the severe ERG defects (Fig. 2B, j-l). In contrast, *w+* and *w+;Gba1b*^*T2A-GAL4*^/+ controls show no obvious signs of neuronal or glial loss (Fig. 3A, c, d, g). However, *w+;anne*^*T2A-GAL4*^/+ flies exhibit a mild photoreceptor and rhabdomere loss along with some vacuolization (Fig. 3A, e, g, red arrowheads and asterisks), but these lesions do not impact ERGs (Fig. 2B, i, l, k). These results clearly indicate that a single-copy loss of *anne* and *Gba1b* first affects glial cells, followed by a progressive neuronal loss.

To assess whether this progressive neuronal loss can be observed in other neuronal populations, we stained the brains of *w+* and *w+;Gba1b*^*T2A-GAL4*^*/+;anne*^*T2A-GAL4*^/+ flies with tyrosine hydroxylase (TH) to assess DA clusters distributed throughout the brain. The *w+;Gba1b*^*T2A-GAL4*^*/+;anne*^*T2A-GAL4*^/+ flies exhibit a ~ 20% reduction in the number of DA neurons in all clusters (Fig. S4). In the PAM cluster, this reduction showed a trend at D15 and was significant by D30 (Fig. S4).

### Partial loss of *anne* and *Gba1b* enhances endo-lysosomal trafficking

Given that *ATP13A2* and *GBA1* converge on regulating lysosome function, we evaluated lysosomal function in *w+;Gba1b*^*T2A-GAL4*^*/+;anne*^*T2A-GAL4*^/+ flies by first assessing lysosomal morphology and abundance. TEM images show that by day 15, there is a significant increase in the number of lysosomes in the photoreceptors of *w+;Gba1b*^*T2A-GAL4*^*/+;anne*^*T2A-GAL4*^/+ flies when compared to controls (Fig. 4A, indicated by yellow asterisks). The size of lysosomes is not increased. This increase in number is not further elevated at day 30 (Fig. 4A, c).

We next assessed the levels of Cathepsin L (CTSL), a lysosomal luminal enzyme. Immunostaining of aged heterozygous fly brains revealed subtle but significantly elevated CTSL levels (Fig. S5A). Moreover, immunostaining for lysobisphosphatidic acid (LBPA), also known as bis-(monoacylglycero)phosphate (BMP), a lysosomal luminal lipid [71], revealed increased BMP levels in *w+;anne*^*T2A-GAL4*^/+ flies, which were further increased in *w+;Gba1b*^*T2A-GAL4*^*/+;anne*^*T2A-GAL4*^/+ flies (Fig. 4B). Together, these findings indicate enhanced lysosomal biogenesis in the double heterozygous mutant flies. Western blot showed that Rab5, an early endosomal marker, was modestly elevated in double heterozygous animals (Fig. S5B, a, b). In addition, we observed mild increases in Dynamin and Rab7 protein levels at D15 (Fig. S5B, a, c, d). These changes in endosomal proteins are consistent with altered endo-lysosomal pathway regulation. In contrast, no significant changes in the levels of downstream targets of Mitf (microphthalmia-associated transcription factor, or transcription factor EB, TFEB) were detected (Fig. S5C), indicating that canonical TFEB-driven biogenesis [72] is not the primary mechanism underneath the increase in lysosome number.

To assess lysosomal acidification, essential for hydrolase activity, we used a dye that labels acidic vesicles, LysoTracker. In aged adult brains (Fig. 4C) and fat bodies (Fig. S5D), LysoTracker staining was significantly reduced in *w+;anne*^*T2A-GAL4*^/+ flies when compared to controls, with a further decrease in *w+;Gba1b*^*T2A-GAL4*^*/+;anne*^*T2A-GAL4*^/+ flies. This reduction was evident from both the decreased number of LysoTracker-positive lysosomes (Fig. 4C, e; Fig. S5D, e) and the diminished LysoTracker intensity (Fig. 4C, f; Fig. S5D, f), indicating impaired lysosomal acidification.

Lysosomal dysfunction can influence mTor (mechanistic Target of rapamycin) signaling and autophagy [12]. To determine whether mTor activity is altered in *w+;Gba1b*^*T2A-GAL4*^*/+;anne*^*T2A-GAL4*^/+ flies, we examined the direct targets of the mTORC1 complex. Specifically, we analyzed S6 kinase (S6K) along with its phosphorylated form, p-S6K. No significant changes in protein levels were observed (Fig. S5E, a). To assess autophagy induction, we measured the levels of the autophagic marker Atg8, which also remained unchanged in *w+;Gba1b*^*T2A-GAL4*^*/+;anne*^*T2A-GAL4*^/+ flies (Fig. S5E, a). Moreover, Western blot analysis of ubiquitin indicates no alterations in ubiquitin-mediated degradation (Fig. S5E, b). These findings show that mTor signaling and autophagy induction are not obviously affected. Hence, lysosomal accumulation likely arises from enhanced endolysosomal flux rather than autophagy induction. In summary, heterozygous loss of *anne* and *Gba1b* drives increased lysosomes, but these lysosomes fail to be acidified properly, culminating in lysosomal dysfunction.

### Loss of *Gba1b* causes GlcCer accumulation in the brain

Given that loss of *Gba1b* impairs glucosylceramide degradation [29, 58], we hypothesized that the heterozygous loss of *anne* and *Gba1b* may alter sphingolipid metabolism. In addition, given that *anne* loss is known to disrupt polyamine metabolism, we also assessed the levels of polyamines [60]. We performed untargeted metabolomics on heads of 1) *w+*, 2) *w+;Gba1b*^*T2A-GAL4*^/+, 3) *w+;anne*^*T2A-GAL4*^/+, and 4) *w+;Gba1b*^*T2A-GAL4*^*/+;anne*^*T2A-GAL4*^/+ flies at days 7, 15, and 30. Across all samples, we detected 780 metabolites, including lipids (Table S2). Principal component analysis (PCA) of these metabolites revealed prominent age-related differences among the four genotypes (Fig. S6A). Further comparisons at each time point identified distinct metabolic changes in double heterozygous animals when compared to controls and single heterozygotes.

**Fig. 5:**
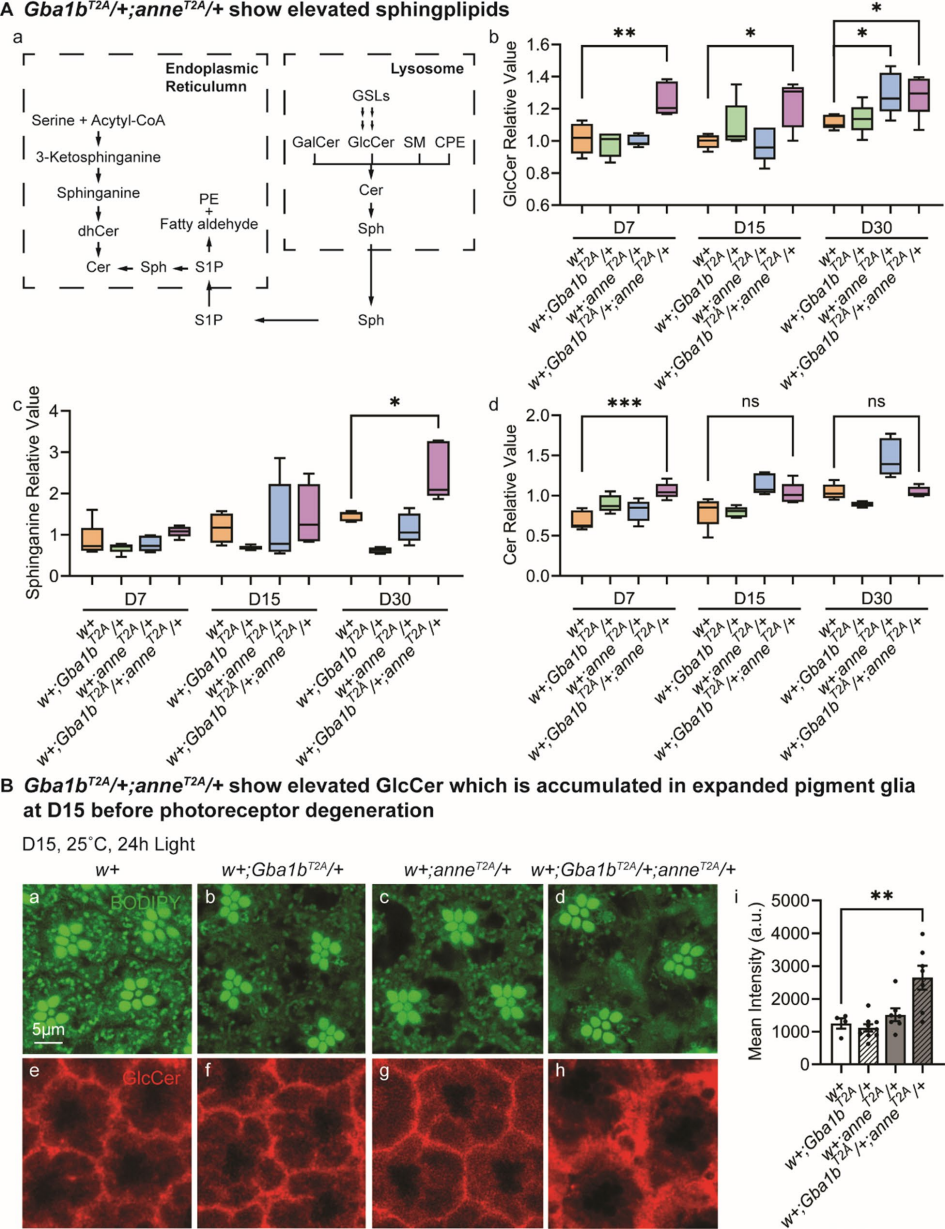
Sphingolipid metabolism is dysregulated in *Gba1b*^*T2A*^*/+;anne*^*T2A*^*/+* flies. (**A**) Sphingolipid metabolism pathways in the ER and lysosome. Cer, ceramide; dhCer, dihydroceramide; GalCer, galactosylceramide; GlcCer, glucosylceramide; CPE, ceramide phosphoethanolamine; SM, sphingomyelin; Sph, sphingosine; S1P, sphingosine-1-phosphate; GSL, glycosphingolipid (**a**). Fold changes in GlcCer (**b**), sphinganine (**c**), and Cer (**d**) levels in the indicated genotypes at days 7, 15, and 30 (*n* = 5). (**B**) BODIPY 493/503 (**a-d**) and glucosylceramide antibody staining of the retina of indicated genotypes (**e-h**) after 15 days of constant light. The mean intensity of glucosylceramide is quantified in i (*n* > 6). Scale bars, 5 μm

Some of the key intermediates of sphingolipid metabolism are shown in Fig. 5A, a. Aberrant profiles were observed in various mutant backgrounds (Fig. 5A, b-d). In double heterozygotes, GlcCer levels were elevated relative to *w+* controls as early as day 7 and these persisted through day 30 (Fig. 5A, b). Notably, *w+;anne*^*T2A-GAL4*^/+ fly heads also showed a significant GlcCer increase by day 30, approaching the levels observed in the double mutants (Fig. 5A, b). Of the > 10 GlcCer species analyzed, more than five were significantly elevated in day 30 double heterozygotes (Fig. S6B). Moreover, immunostaining for GlcCer of 15-day-old fly retinas showed that the GlcCer levels in the double heterozygous flies were significantly elevated and accumulated in swollen glia (Fig. 5B). Additionally, sphinganine levels were significantly increased in day 30 double heterozygotes compared to controls and single heterozygotes (Fig. 5A, c), suggesting an upregulation of the de novo ceramide biosynthesis pathway in the endoplasmic reticulum (Fig. 5A, a). Ceramide levels showed a modest increase in double heterozygotes at day 7, without further age-dependent accumulation (Fig. 5A, d). Interestingly, *w+;anne*^*T2A-GAL4*^/+ flies also exhibited increased ceramide levels at days 15 and 30 (Fig. 5A, d). In summary, these data document a dysregulation in sphingolipid metabolism which is consistent with a defect in the breakdown of GlcCer and a dysregulation of the *de novo* ceramide synthesis.

Polyamine profiling revealed a general age-dependent increase in polyamine levels. However, no distinct genotype-specific patterns were observed (Fig. S6C, a, b). Notably, spermidine levels were selectively reduced in double heterozygotes by day 30 (Fig. S6C, b, c), while spermine levels remained unchanged (Fig. S6C, b, d). This reduction in spermidine was not detected at day 7 or 15, suggesting that dysregulation emerges later during the progression of neurodegeneration and is not a primary driver.

Although PCA revealed clear age-dependent shifts in metabolite composition, the genotype explained only a small fraction of total variance. This was not unexpected given that all genotypes analyzed were heterozygous for loss-of-function alleles, which are unlikely to produce widespread metabolic remodeling. Instead, PCA performed within individual ages revealed partially overlapping genotype clusters, suggesting subtle but biologically meaningful metabolic differences. Our targeted analyses indicate that these differences are most obvious for specific sphingolipid species, particularly GlcCer and sphinganine, rather than global alterations across the metabolome. Thus, we propose that changes in a limited set of key lipid metabolites, rather than broad metabolic reprogramming, drive the observed neurodegenerative phenotypes.

### Improving lysosomal function rescues neurodegenerative phenotypes in *Gba1b*^***T2A-GAL4***^*/+;anne*^*T2A-GAL4*^*/+ ***flies**

**Fig. 6:**
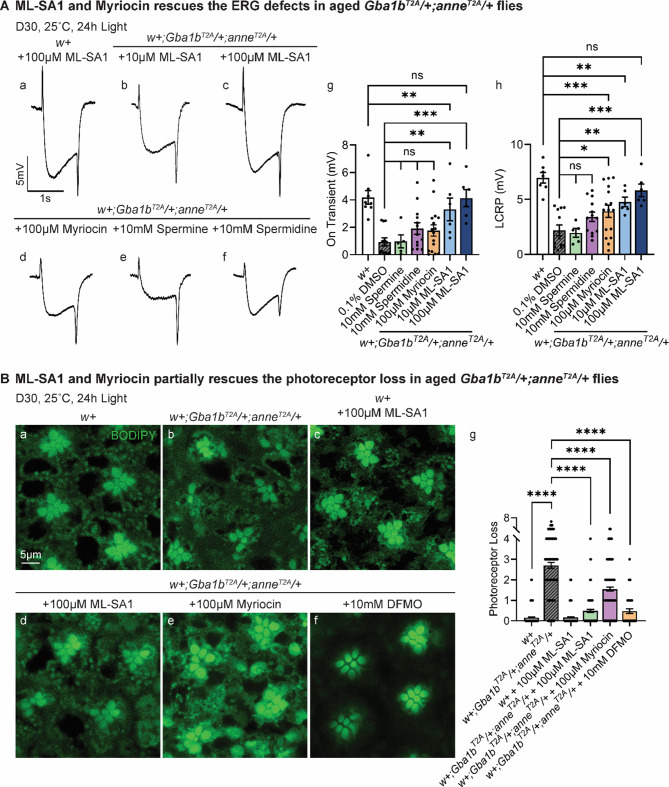
Boosting lysosomal functions rescues the neurodegeneration in aged *Gba1b*^*T2A*^*/+;anne*^*T2A*^/+ flies. (**A**) ERG recordings of flies of the indicated genotypes treated with ML-SA1, myriocin, spermine, or spermidine for 30 days in constant light. The reduction in the LCRPs and on/off-transients in *w+;Gba1b*^*T2A*^*/+;anne*^*T2A*^/+ flies was partially rescued with ML-SA1 and myriocin feeding. The ERG LCRP and on-transient amplitudes are quantified in g and h (*n* > 6). Error bars represent SEM; **p < 0.01. (**B**) BODIPY 493/503 staining of the retina of indicated genotypes with drug treatment after 30 days of constant light. The loss of photoreceptors in *w+;Gba1b*^*T2A*^*/+;anne*^*T2A*^/+ flies was partially rescued with ML-SA1, myriocin, and DFMO feeding. The number of photoreceptors is quantified in g (*n* > 10). Scale bars, 5 μm

Given the impairment in lysosomal acidification, we aimed to restore lysosomal function and assessed both physiological function (via ERGs) and tissue morphology (retina structure). ML-SA1, a specific agonist of the lysosomal TRPML1 channel, promotes lysosomal trafficking, enhances lysosomal biogenesis, and stimulates autophagic flux through TFEB activation [73]. Feeding *w+;Gba1b*^*T2A-GAL4*^*/+;anne*^*T2A-GAL4*^/+ flies ML-SA1 resulted in a dose-dependent rescue of the ERG defects observed in 30-day-old flies (Fig. 6A). Flies treated with 10 µM ML-SA1 exhibited a partial but significant improvement in LCRPs (Fig. 6A, b, g, h), whereas those treated with 100 µM ML-SA1 showed a complete rescue of the on/off transients and LCRP defects (Fig. 6A, c, g, h).

Metabolomics analysis revealed that sphingolipid metabolism is altered in aged *w+;Gba1b*^*T2A-GAL4*^*/+;anne*^*T2A-GAL4*^/+ flies, accompanied by some dysregulation of polyamine levels. To assess the impact of these metabolic changes, we treated flies with a drug that inhibits sphingolipid synthesis and supplemented the food with polyamines and difluoromethylornithine (DFMO). Treatment with myriocin, a compound that reduces *de novo* synthesis of ceramide and its derivatives, partially but significantly restored ERG responses in 30-day-old double heterozygous fly eyes (Fig. 6A, d, g, h). In contrast, supplementation with polyamines, including 10 mM spermine or 10 mM spermidine, failed to significantly improve ERG defects in aged *w+;Gba1b*^*T2A-GAL4*^*/+;anne*^*T2A-GAL4*^/+ flies (Fig. 6A, e, f, g, h). Interestingly, inhibiting polyamine synthesis utilizing 10 mM DFMO, a drug that reduces the activity of ornithine decarboxylase, an enzyme that is required for polyamine biosynthesis [74–76] (Fig. S6C, a), partially rescued the climbing and nearly fully rescued the ERG defects in 30-day-old flies (Fig. S7).

To assess photoreceptor morphology, we stained retinas with BODIPY 493/503, which labels neutral lipids and highlights rhabdomeres as well as other retinal structures. BODIPY staining revealed a partial but significant suppression of photoreceptor loss in double heterozygous mutant flies treated with 100 µM ML-SA1, 100 µM myriocin, and 10 mM DFMO (Fig. 6B). Taken together, these findings demonstrate that enhancing lysosomal function can partially rescue the neurodegenerative phenotypes in *w+;Gba1b*^*T2A-GAL4*^*/+;anne*^*T2A-GAL4*^/+ flies. Cer/GlcCer accumulation is a driver of the neurodegenerative phenotypes, and polyamine imbalance also plays a role in the neurodegenerative process.

### Double heterozygous loss-of-function variants in *GBA1* and *ATP13A2* in individuals with PD

To translate our findings, we interrogated available human genetic data and explored whether digenic variation in *GBA1* and *ATP13A2* may interact to drive PD in humans. We first interrogated our local collection of PD cases with genome sequencing from Baylor College of Medicine (*n* = 149; mean age = 65; 37% female). Overall, 9 cases were carriers of established *GBA1* risk alleles for PD, including a single case that was double heterozygous for *GBA1* (p.T408M) as well as a variant in *ATP13A2* (p.R370W). This individual had onset of unilateral rest tremor and gait/balance difficulty in his 60s, a positive dopamine transporter scan, and was levodopa responsive, consistent with typical PD. Family history was notable for PD in the individual’s father with disease onset in his 80s. Besides the *GBA1* and *ATP13A2* variants, there were no other rare variant PD risk alleles detected in the individual’s genome.

**Fig. 7:**
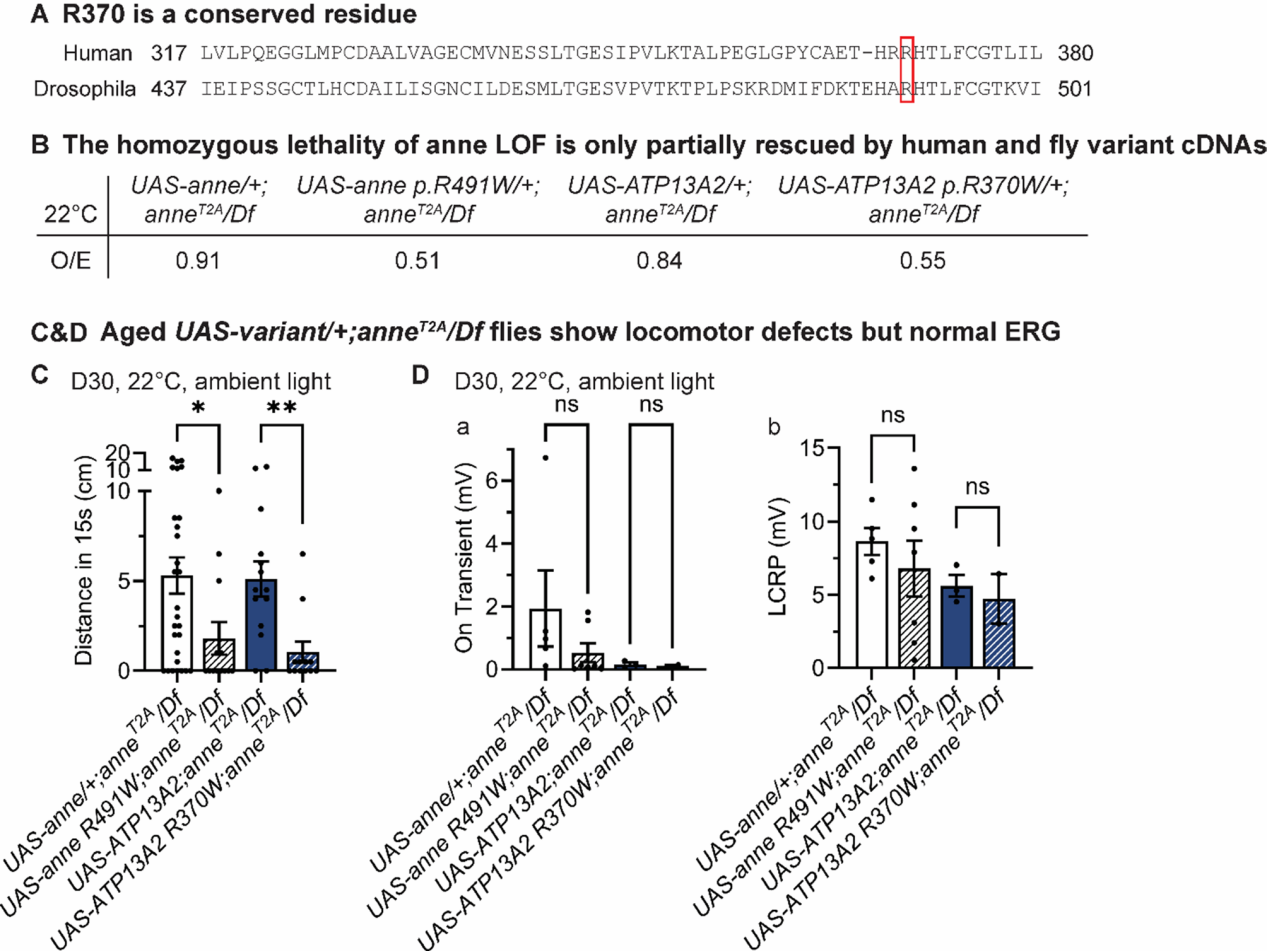
*anne* *p.R491W* is a weak loss of function allele. (**A**) Amino acid conservation of a portion of the fly and human ATP13A2 proteins. (**B**) The rescue ability of reference and variant fly and human cDNAs. *UAS-anne* or *UAS-ATP13A2* Ref almost completely rescues the lethality of *anne* LoF mutants. *UAS-anne p.R491W* or *UAS-ATP13A2 p.R370W* partially rescues lethality (*n* > 100). (**C**) Climbing analysis of the flies of the indicated genotypes after 30 days of ambient light. *UAS-anne p.R491W/+;anne*^*T2A*^*/Df* and *UAS-ATP13A2 p.R370W/+;anne*^*T2A*^*/Df* flies exhibit climbing defects. Flies were raised at 22 °C (*n* > 20). (**D**) Analysis of ERG recordings of flies of the indicated genotypes after 30 days of ambient light. *UAS-anne p.R491W/+;anne*^*T2A*^*/Df* and *UAS-ATP13A2 p.R370W/+;anne*^*T2A*^*/Df* flies show no changes in LCRP or on-transient amplitudes compared to *UAS-anne/+;anne*^*T2A*^*/Df* and *UAS-ATP13A2/+;anne*^*T2A*^*/Df* flies (*n* > 6). Error bars represent SEM; *p < 0.05, **p < 0.01

The *GBA1* p.T408M variant is an established, mild loss-of-function allele that reduces GCase activity to approximately 60% and has been associated with a 1.4 to 5-fold increased risk of PD [77–80]. However, the *ATP13A2* p.R370W variant has not been previously linked to Kufor-Rakeb syndrome or parkinsonism. In public databases of control individuals, *ATP13A2* p.R370W is rare (freq. = 4.21x10^−5^) (gnomAD), and is predicted to be likely damaging based on multiple bioinformatic prediction tools (CADD score = 24.9) [81]. Arginine 370 falls within the cytosolic ATP-binding domain of *ATP13A2,* and this residue is conserved in Drosophila *anne*, where it corresponds to R491 (Fig. 7A). To determine whether *anne* p.R491W is a loss-of-function mutation, we expressed the variant using the *T2A-GAL4/Df > UAS-anne p.R491W* system. While lethality in *anne*^*T2A-GAL4*^*/Df* flies was fully rescued by wild-type *anne* cDNAs (Fig. 1B, b), the R491W variant only achieved ~50% rescue (Fig. 7B). A similar partial rescue was observed using the human *ATP13A2* p.R370W cDNA (Fig. 7B). We then evaluated the motor function and retinal physiology of flies rescued with the variant proteins. Climbing assays revealed that 30-day-old *UAS-ATP13A2 p.R370W/+;anne*^*T2A-GAL4*^*/Df* and *UAS-anne p.R491W/+;anne*^*T2A-GAL4*^*/Df* flies exhibited a significantly reduced locomotor activity (Fig. 7C). However, their ERG responses were not significantly affected (Fig. 7D). These findings indicate that *ATP13A2* p.R370W is a partial loss-of-function allele.

In order to better estimate how frequently digenic variants in *GBA1* and *ATP13A2* are found in PD, we interrogated clinical exome and genome sequencing from 15,881 individuals with PD from the Global Parkinson’s Genetics Program (GP2) [44, 47] (Tables S3 and S4). *GBA1* risk variants were identified in 1292 individuals. Among those 1292 *GBA1*-PD cases with heterozygous *GBA1* variants, we identified 5 additional cases with three different heterozygous likely pathogenic *ATP13A2* variants (p.Y1020Tfs*3, p.Q635*, c.477+2T > G) (Table S3) (freq. = 0.39%). In addition, we discovered 2 more individuals with heterozygous *ATP13A2* variants among 210 GP2 cases recruited with previously known *GBA1*-PD, both with *GBA1* p.N409S and *ATP13A2* p.Y1020Tfs*3. Lastly, to estimate the population background frequency of digenic putative pathogenic variants in *GBA1* and *ATP13A2*, we interrogated genome sequence data from the United Kingdom Biobank (UKBB). Among 25,869 heterozygous unaffected carriers of *GBA1* risk alleles (without known PD), we found 17 individuals also carrying a likely pathogenic variant in *ATP13A2* (freq. = 0.06%). Our results are therefore suggestive of a higher rate of heterozygous mutations in *ATP13A2* among individuals with *GBA1-*PD.

## Discussion

The aim of our study was to test the hypothesis that partial loss-of-function in LSD genes may modify *GBA1*-PD penetrance. We observed that partial loss of *Gba1b* and *anne*, the fly orthologs of *GBA1* and *ATP13A2* in humans, synergistically promote neurodegeneration by affecting lysosomal function and disrupting sphingolipid metabolism. These findings provide experimental support for the hypothesis that *GBA1* penetrance is influenced by additional genetic modifiers. Given that *anne* is expressed in neurons and *Gba1b* in glia throughout development and adulthood, these phenotypes result from an aberrant neuron-to-glia crosstalk.

### Increased GlcCer production in neurons affects glial morphology prior to neuronal insults

The time course and spatial progression of neurodegeneration in our model highlights a critical role for neuron-glia crosstalk in maintaining metabolic homeostasis. The first severe phenotype that we observed is an obvious disruption of the morphology of glia that surround the photoreceptors. This disruption was associated with a subtle loss of function in ERGs as well as a subtle neuronal loss, suggesting that glia are early drivers in the pathology (Fig. 3). This finding is consistent with observations from zebrafish and mouse models, where microglial activation and astrocytic dysfunction occur prior to neuronal degeneration in *GBA*-deficient contexts [82–84]. Our previous studies have established the importance of neuron-to-glia lipid trafficking, wherein neurons produce GlcCer that is released for uptake in glia upon a glial signal: secretion of TGF-β in vertebrate cell cultures or Dpp/Daw in Drosophila. When glial *Gba1b* function is lost, GlcCer accumulates, first in glia, then in neurons [29]. However, *w+;Gba1b*^*T2A*^/+ flies showed no overt neurological defects and no increase in GlcCer (Figs. 2, 3A, 5), suggesting that one wild type *Gba1b* allele is sufficient for proper glial and neuronal function. However, loss of a single copy of *ATP13A2/anne* in the *w+;Gba1b*^*T2A*^/+ flies may lead to increased GlcCer production, which is exported from neurons to glia, where it may overwhelm the glial degradative capacity in heterozygous cells and cause cellular toxicity. Consistent with this hypothesis, lipidomic analyses revealed that GlcCer in *w+;Gba1b*^*T2A*^*/+;anne*^*T2A*^/+ flies is elevated when compared to *w+* or *Gba1b* or *anne* single heterozygotes (Fig. 5A, b and Fig. S6B). This is accompanied by a progressive increase in sphinganine levels (Fig. 5A, c), a product of the *de novo* ceramide synthesis pathway. Interestingly, ceramide synthesis has been reported to be upregulated in the cingulate gyrus of PD patients carrying a *GBA1* mutation [85]. Sphinganine accumulation has also been reported in the substantia nigra of MPTP (1-methyl-4-phenyl-1,2,3,6-tetrahydropyridine)-treated mice [86], a PD model in which this neurotoxin destroys nigrostriatal dopaminergic neurons [87]. Similarly, in human studies, increased ceramide levels have been detected in plasma, serum, cerebrospinal fluid, and brain tissue of PD patients [88–94]. Our data documents an early ceramide accumulation starting at day 7 in double heterozygous flies (Fig. 5A, d). Given the well-established neurotoxicity of elevated ceramide levels in various fly PD models, including loss of *PLA2G6*, synuclein overexpression, *VPS35* loss [95], and *Pink1* loss [96, 97], these findings support a pathological role for sphingolipid imbalance. Importantly, treatment with myriocin partially rescues ERG defects and retinal degeneration in double heterozygotes (Fig. 6), implicating GlcCer accumulation and broader sphingolipid dysregulation as key drivers of glial and neuronal dysfunction.

Interestingly, the gene required for GlcCer synthesis is expressed in neurons but not in glia. The presence of GlcCer in glia therefore depends on export and subsequent uptake of exosomes from neurons. Indeed, immunostaining of 15-day-old double heterozygous retina demonstrated an accumulation of GlcCer in the pigment glia, but not in neurons (Fig. 5B). Notably, the GlcCer immunostaining also revealed a glial expansion and vacuolization (Fig. 5B), consistent with the TEM data (Fig. 3) that glial morphological changes and dysfunction occur prior to photoreceptor degeneration. Accumulation of GlcCer persisted on day 30 in fly retina immunostaining, when severe neurodegeneration was observed. At this stage, GlcCer was detected in both pigment glia and the photoreceptors in the *w+;Gba1b*^*T2A*^*/+;anne*^*T2A*^/+ retina, suggesting a potential defect in neuron-to-glia GlcCer transport (Fig. S8). In the absence of neuronal activity, neurons do not produce GlcCer and we have previously shown that neuronal activity strongly promotes GlcCer production using several stimulation paradigms, including light [29]. We therefore tested whether reducing neuronal activity could mitigate degeneration in *w+;Gba1b*^*T2A*^*/+;anne*^*T2A*^/+ flies. Indeed, *w+;Gba1b*^*T2A*^*/+;anne*^*T2A*^/+ flies reared in constant darkness to eliminate light-dependent photoreceptor activity maintain robust ERG responses even at day 45 (Fig. S9). This supports a model in which *w+;Gba1b*^*T2A*^*/+;anne*^*T2A*^/+ neurons produce too much GlcCer leading to an overload of partially compromised glial lysosomes with GlcCer, leading to dysfunctional glial cells with morphological defects that are already obvious at day 15 (Fig. 3).

### ATP13A2-associated polyamine changes contribute to neurodegeneration

Complete loss of *ATP13A2* disrupts lysosomal polyamine export, leading to intralysosomal accumulation and a net decrease in total cellular polyamines compared to controls [60]. Whether heterozygous loss yields a similar polyamine deficit remains unknown. In mice, heterozygosity for an *ATP13A2* knockout allele triggers microgliosis and astrocytosis in cortex and hippocampus by 18 months [98]. In Drosophila, *anne* single heterozygous mutants exhibit mild photoreceptor dysfunction [99], and *anne* knockdown in α-synuclein-expressing animals impairs locomotion [100]. Consistent with these observations we observe modest age-dependent locomotor deficits (Fig. 2A, b) and photoreceptor degeneration (Fig. 3A, e, g) in day-30 *w+;anne*^*T2A*^/+ flies. Whether these phenotypes arise from altered polyamine handling is unclear.

Metabolomic profiling of *w+;anne*^*T2A*^/+ heads revealed no significant changes in polyamine levels. In contrast, *w+;Gba1b*^*T2A*^*/+;anne*^*T2A*^/+ flies exhibited reduced levels of spermidine and acetyl-spermidine at day 30 (Fig. S6C, a-c), a time point at which significant neurodegeneration is already evident. However, dietary polyamine supplementation only modestly improved neurodegeneration (Fig. 6A). On the other hand, DFMO showed rescue in climbing and ERG assays as well as photoreceptor loss (Figs. S7 and 6B), suggesting that inhibiting polyamide synthesis can partially reverse the neurodegenerative phenotypes. This is surprising as we did not detect a significant increase in polyamine levels in our metabolomics assay. However, the number of polyamines we detected using our panel was quite limited. Since ATP13A2 functions in exporting polyamine into the cytosol, partial loss-of-function of ATP13A2 may result in a compensatory elevation in polyamine synthesis in the cytosol. Excess cytosolic polyamine levels can lead to toxicity [101], and DFMO is known to reduce the cytoplasmic levels of polyamines [74–76]. Detailed mechanisms may require further investigation, but the DFMO data indicates that polyamine imbalance plays a role in driving neurodegeneration.

### Neuronal endo-lysosomal acidification defects may drive excess GlcCer export from neurons, thereby promoting both glial and neuronal dysfunction

ATP13A2 has been shown to be a cotransporter of K^+^ and H^+^ across the late-endosomal/lysosomal membrane [61]. Consistent with this observation, we found that lysosomal acidification is modestly compromised in 15-day-old *w+;anne*^*T2A*^/+ flies. However, lysosomal acidification is severely affected in w*+;Gba1b*^*T2A*^*/+;anne*^*T2A*^/+ animals (Fig. 4C). Moreover, at day 15, photoreceptors of *w+;Gba1b*^*T2A*^*/+;anne*^*T2A*^/+ flies exhibit an increase in lysosome number (Fig. 4A), and by day 30, we observe aberrant lysosomes (Fig. 3A). Indeed, TEM images do not reveal obvious lysosomal enlargements, but many of the phenotypes we documented mirrored typical changes seen in human LSDs, such as increased lysosome number due to enhanced biogenesis [102, 103], the presence of electrodense, partially degraded material, and multi-membrane structures [104, 105]. Although we did not detect elevated TFEB-mediated biogenesis (Fig. S5D), we found that several endosomal proteins, Rab5, Rab7, and Dynamin (Fig. S5B), are modestly upregulated. In addition, the levels of two lysosomal markers (CTSL and BMP) are also upregulated (Figs. 4B, S6A). These data are consistent with an increase in endocytic flux.

Most LSD phenotypes arise from secondary storage and trafficking defects rather than the primary enzyme deficiency [73, 106]. Therefore, boosting lysosomal function and membrane trafficking can clear aggregated compartments and confer protection. For example, in Niemann-Pick C models, activation of the lysosomal Ca^2+^ channel TRPML1 enhances exocytosis of lysosomes and clears cholesterol-filled lysosomes [73]. Similarly, ML-SA1 treatment in our *w+;Gba1b*^*T2A*^*/+;anne*^*T2A*^/+ model nearly fully rescued photoreceptor function and ERG defects (Fig. 6). These data suggest that enhancing lysosomal exocytosis and membrane trafficking is sufficient to reverse neurodegenerative phenotypes.

Taken together, our data suggest that loss of *anne* disrupts neuronal GlcCer trafficking, augmenting its transfer to glia in an activity-dependent manner. While lysosomal dysfunction clearly contributes, the mechanistic link between impaired acidification and GlcCer accumulation remains to be defined. GlcCer is synthesized from ceramide in the neuronal Golgi [107–110] and trafficked to the plasma membrane [111]. Once GlcCer reaches the plasma membrane, it can be internalized through endocytic pathways [112, 113] and sorted into multivesicular bodies for exosomal release [29]. Photoreceptor activity drives extensive membrane remodeling [114–117], which may underlie high rates of GlcCer turnover and transfer to glia. Recent work implicates the Commander and the retromer complexes, which are required for endosomal sorting, in modulating glucocerebrosidase (GCase) activity and PD risk, further linking endo-lysosomal trafficking to GlcCer metabolism [95, 118].

During endolysosomal maturation, progressive luminal acidification orchestrates cargo sorting and membrane identity. Disruption of this pH gradient, through V-ATPase inhibition or lipid accumulation, impairs sorting complexes and intraluminal vesicle (ILV) formation, yielding aberrant multivesicular bodies and increased ILV/exosomal release of undegraded cargo [119–121]. Since BMP marks ILVs within both lysosomes and the late endosomal MVBs [122, 123], the increased BMP staining we observe may also reflect elevated ILV/exosome production (Fig. 4B). Moreover, impaired lysosomal function was associated with EV biogenesis [124, 125]. Thus, loss of *anne* likely impairs late-endosomal/lysosomal pH, promoting GlcCer transport via exosomes because of defective endo-lysosomal trafficking.

### Relevance for GBA1-PD

*GBA1* causes an approximately 5-fold increased risk of PD [16, 126]. Mutations causing more severe loss of GCase enzymatic activity are associated with increased penetrance for PD and greater risk of PD related cognitive impairment, supporting a loss-of-function mechanism [127]. Whereas Gaucher’s disease requires a loss of more than 85% of the GCase activity [128], increased PD risk maybe seen with as little as 30% reduction, such as in heterozygous individuals with the *GBA1* p.T408M variant [129, 130]. Besides, *GBA1* PD risk variants have been identified in other genes that cause LSDs, including *ARSA, GALC,* and *SMPD1*[3]. A burden of rare damaging variants among LSD genes has also been associated with PD risk, and up to one-fifth of PD cases may have multiple such variants [30, 131]. These results are consistent with a possible oligogenic model in which genetic variants in other genes may interact with *GBA1* heterozygosity to modify PD penetrance. In our local PD sample, we identified an individual with *GBA1-*PD who was also a carrier of a novel loss-of-function variant in *ATP13A2* (p.R370W) (Fig. 7). Additional data mining in the GP2 cohort identified additional PD cases with double heterozygous variants in *GBA1* and *ATP13A2*, consistent with a digenic model (Table S3). Our results are consistent with a prior study highlighting an increased frequency of *ATP13A2* heterozygous variants among individuals with PD [132].

## Conclusions

Our findings demonstrate that cell-type–specific lysosomal gene haploinsufficiency cooperatively impairs lysosomal acidification and elevates glucosylceramide, initiating a neuron-glia cross-talk failure and neurodegeneration in Drosophila. These results substantiate a mechanistic framework whereby subtle, combined lysosomal defects—as seen in human heterozygous carriers of *GBA1* and *ATP13A2* variants—drive PD pathology. The reversal of these defects by ML-SA1, myriocin, and DFMO underscores the translational potential of targeting lysosomal membrane trafficking, sphingolipid metabolism and polyamine imbalance for early therapeutic intervention in polygenic neurodegeneration.

## Electronic supplementary material

Below is the link to the electronic supplementary material.

Supplementary Material 1

Supplementary Material 2

Supplementary Material 3

Supplementary Material 4

Supplementary Material 5

## Data Availability

Data used in the preparation of this article were obtained from the Global Parkinson’s Genetics Program (GP2; https://gp2.org). GP2 is funded by the Aligning Science Across Parkinson’s (ASAP) initiative and implemented by The Michael J. Fox Foundation for Parkinson’s Research (https://gp2.org). For a complete list of GP2 members see https://gp2.org. All fly strains are sent to the Bloomington Drosophila Stock Center. Further information and requests for other resources and reagents should be directed to and will be fulfilled by the Lead Contact, Hugo J. Bellen (hbellen@bcm.edu).

## Abbreviations

BAC: bacterial artificial chromosome
BMP: Bis(monoacylglycero)phosphate
CMA: Chaperone-mediated autophagy
CNS: central nervous system
CTSL: Cathepsin L
DA: dopaminergic
DFMO: difluoromethylornithine
ERG: Electroretinogram
GD: Gaucher disease
GlcCer: Glucosylceramide
GCase: Glucocerebrosidase
GWAS: Genome‑wide association study
ILV: Intraluminal vesicle
LSD: Lysosomal storage disorder
MVB: Multivesicular body
PCA: Principal component analysis
PD: Parkinson’s disease
S6K: S6 kinase
TEM: Transmission electron microscopy
TFEB: transcription factor EB
TH: tyrosine hydroxylase

## References

[1] Spillantini MG, Schmidt ML, Lee VMY, Trojanowski JQ, Jakes R, Goedert M. α-synuclein in Lewy bodies. Nat. 1997, Aug 28;388(6645):839–40. 10.1038/42166 9278044

[2] Fearnley JM, Lees AJ. Ageing and Parkinson’s disease: substantia nigra regional selectivity. Brain. 1991, Oct 1;114(5):2283–301. 10.1093/brain/114.5.2283 1933245

[3] Ye H, Robak LA, Yu M, Cykowski M, Shulman JM. Genetics and pathogenesis of Parkinson’s syndrome. Annu Rev Pathol. 2023, Jan 24;18:95–121. 10.1146/annurev-pathmechdis-031521-034145 PMC10290758 36100231

[4] Nalls MA, Blauwendraat C, Vallerga CL, Heilbron K, Bandres-Ciga S, Chang D, et al. Identification of novel risk loci, causal insights, and heritable risk for Parkinson’s disease: a meta-analysis of genome-wide association studies. Lancet Neurol. 2019, Dec 1;18(12):1091–102. 10.1016/S1474-4422(19)30320-5 PMC8422160 31701892

[5] Kalia LV, Lang AE. Parkinson’s Disease. Lancet. 2015, Aug 29;386(9996):896–912. 10.1016/S0140-6736(14)61393-3 25904081

[6] Moors T, Paciotti S, Chiasserini D, Calabresi P, Parnetti L, Beccari T, et al. Lysosomal dysfunction and α-synuclein aggregation in Parkinson’s disease: diagnostic links. Mov Disord. 2016, Jun 1;31(6):791–801. 10.1002/mds.26562 26923732

[7] Wong YC, Krainc D. Lysosomal trafficking defects link Parkinson’s disease with Gaucher’s disease. Mov Disord. 2016, Nov 1;31(11):1610–18. 10.1002/mds.26802 PMC5957289 27619775

[8] Coukos R, Krainc D. Key genes and convergent pathogenic mechanisms in Parkinson disease. Nat Rev Neurosci. 2024, Jun;25(6):393–413. 10.1038/s41583-024-00812-2 38600347

[9] Hull A, Atilano ML, Gergi L, Kinghorn KJ. Lysosomal storage, impaired autophagy and innate immunity in Gaucher and Parkinson’s diseases: insights for drug discovery. Philos Trans R Soc B Biol Sci. 2024, Apr 8;379(1899):20220381. 10.1098/rstb.2022.0381 PMC10874704 38368939

[10] Blumenreich S, Barav OB, Jenkins BJ, Futerman AH. Lysosomal storage disorders shed light on lysosomal dysfunction in Parkinson’s disease. Int J Mol Sci. 2020, Jul 14;21(14):4966. 10.3390/ijms21144966 PMC7404170 32674335

[11] Xu H, Ren D. Lysosomal physiology. Annu Rev Physiol. 2015, Feb 1;2015:77:57–80. 10.1146/annurev-physiol-021014-071649 PMC4524569 25668017

[12] Li P, Gu M, Xu H. Lysosomal ion channels as decoders of cellular signals. Trends Biochem Sci. 2019, Feb 1;44(2):110–24. 10.1016/j.tibs.2018.10.006 PMC6340733 30424907

[13] Mazzulli JR, Xu YH, Sun Y, Knight AL, McLean PJ, Caldwell GA, et al. Gaucher disease glucocerebrosidase and α-synuclein form a bidirectional pathogenic loop in synucleinopathies. Cell. 2011, Jul 8;146(1):37–52. 10.1016/j.cell.2011.06.001 PMC3132082 21700325

[14] Cuervo AM, Stafanis L, Fredenburg R, Lansbury PT, Sulzer D. Impaired degradation of mutant α-synuclein by chaperone-mediated autophagy. Sci. 2004, Aug 27;305(5688):1292–95. 10.1126/science.1101738 15333840

[15] Aharon-Peretz J, Rosenbaum H, Gershoni-Baruch R. Mutations in the glucocerebrosidase gene and Parkinson’s disease in Ashkenazi Jews. N Engl J Med. 2004, Nov 4;351(19):1972–77. 10.1056/NEJMoa033277 15525722

[16] Sidransky E, Nalls MA, Aasly JO, Aharon-Peretz J, Annesi G, Barbosa ER, et al. Multicenter analysis of glucocerebrosidase mutations in Parkinson’s disease. N Engl J Me. 2009, Oct 22;361(17):1651–61. 10.1056/NEJMoa0901281 PMC2856322 19846850

[17] Nalls MA, Pankratz N, Lill CM, Do CB, Hernandez DG, Saad M, et al. Large-scale meta-analysis of genome-wide association data identifies six new risk loci for Parkinson’s disease. Nat Genet. 2014, Sep 1;46(9):989–93. 10.1038/ng.3043 PMC4146673 25064009

[18] Deng H, Xiu X, Jankovic J. Genetic convergence of Parkinson’s disease and lysosomal storage disorders. Mol Neurobiol. 2015, Jun 1;51(3):1554–68. 10.1007/s12035-014-8832-4 25099932

[19] Wong K, Sidransky E, Verma A, Mixon T, Sandberg GD, Wakefield LK, et al. Neuropathology provides clues to the pathophysiology of Gaucher disease. Mol Genet Metab. 2004, Jul 1;82(3):192–207. 10.1016/j.ymgme.2004.04.011 15234332

[20] Westenberger A, Skrahina V, Usnich T, Beetz C, Vollstedt EJ, Laabs BH, et al. Relevance of genetic testing in the gene-targeted trial era: the rostock Parkinson’s disease study. Brain. 2024, Aug 1;147(8):2652–67. 10.1093/brain/awae188 PMC11292909 39087914

[21] Cook L, Verbrugge J, Schwantes-An TH, Schulze J, Foroud T, Hall A, et al. Parkinson’s disease variant detection and disclosure: PD GENEration, a North American study. Brain. 2024, Aug 1;147(8):2668–79. 10.1093/brain/awae142 PMC11292896 39074992

[22] Lesage S, Anheim M, Condroyer C, Pollak P, Durif F, Dupuits C, et al. Large-scale screening of the Gaucher’s disease-related glucocerebrosidase gene in europeans with Parkinson’s disease. Hum Mol Genet. 2011, Jan 1;20(1):202–10. 10.1093/hmg/ddq454 20947659

[23] Neumann J, Bras J, Deas E, O’sullivan SS, Parkkinen L, Lachmann RH, et al. Glucocerebrosidase mutations in clinical and pathologically proven Parkinson’s disease. Brain. 2009, Jul 1;132(7):1783–94. 10.1093/brain/awp044 PMC2702833 19286695

[24] De Marco E V, Annesi G, Tarantino P, Rocca FE, Provenzano G, Civitelli D, et al. Glucocerebrosidase gene mutations are associated with Parkinson’s disease in Southern Italy. Mov Disord. 2008, Feb 15;23(3):460–63. 10.1002/mds.21892 18074383

[25] Yu Z, Wang T, Xu J, Wang W, Wang G, Chen C, et al. Mutations in the glucocerebrosidase gene are responsible for Chinese patients with Parkinson’s disease. J Hum Genet. 2015, Feb 1;60(2):85–90. 10.1038/jhg.2014.110 25518742

[26] Rossi M, Schaake S, Usnich T, Boehm J, Steffen N, Schell N, et al. Classification and genotype-phenotype relationships of GBA1 variants: MDSGene systematic review. Mov Disord. 2025, Apr 1;40(4):605–18. 10.1002/mds.30141 PMC12006889 39927608

[27] Sanchez-Martinez A, Beavan M, Gegg ME, Chau KY, Whitworth AJ, Schapira AHV. Parkinson disease-linked GBA mutation effects reversed by molecular chaperones in human cell and fly models. Sci Rep. 2016, Aug 19;6(1):1–12. 10.1038/srep31380 PMC4990939 27539639

[28] Brady RO. The sphingolipidoses. N Engl J Med. 1966, Aug 11;275(6):312–18. 10.1056/NEJM196608112750606 5940695

[29] Wang L, Lin G, Zuo Z, Li Y, Byeon SK, Pandey A, et al. Neuronal activity induces glucosylceramide that is secreted via exosomes for lysosomal degradation in glia. Sci Adv. 2022, Jul 15;8(28):3326. 10.1126/sciadv.abn3326 PMC9278864 35857503

[30] Robak LA, Jansen IE, van RJ, Uitterlinden AG, Kraaij R, Jankovic J, et al. Excessive burden of lysosomal storage disorder gene variants in Parkinson’s disease. Brain. 2017, Dec 1;140(12):3191–203. 10.1093/brain/awx285 PMC5841393 29140481

[31] Kanca O, Zirin J, Garcia-Marques J, Knight SM, Yang-Zhou D, Amador G, et al. An efficient CRISPR-based strategy to insert small and large fragments of DNA using short homology arms. Elife. 2019, Nov 1;8:e51539. 10.7554/eLife.51539 PMC6855806 31674908

[32] Kanca O, Zirin J, Hu Y, Tepe B, Dutta D, Lin WW, et al. An expanded toolkit for Drosophila gene tagging using synthesized homology donor constructs for CRISPR-mediated homologous recombination. Elife. 2022, Jun 20;11:e76077. 10.7554/eLife.76077 PMC9239680 35723254

[33] Venken KJT, Carlson JW, Schulze KL, Pan H, He Y, Spokony R, et al. Versatile P[Acman] BAC libraries for transgenesis studies in Drosophila melanogaster. Nat Methods. 2009, May 24;6(6):431–34. 10.1038/nmeth.1331 PMC2784134 19465919

[34] Hu Y, Flockhart I, Vinayagam A, Bergwitz C, Berger B, Perrimon N, et al. An integrative approach to ortholog prediction for disease-focused and other functional studies. BMC bioinformatics. 2011, Aug 31;12:357. 10.1186/1471-2105-12-357 PMC3179972 21880147

[35] Platt FM, D’Azzo A, Davidson BL, Neufeld EF, Tifft CJ. Lysosomal storage diseases. Nat Rev Dis Primers. 2018, Oct 1;4(1):27. 10.1038/s41572-018-0025-4 30275469

[36] Yu M, Ye H, De-Paula RB, Mangleburg CG, Wu T, Lee T V, et al. Functional screening of lysosomal storage disorder genes identifies modifiers of alpha-synuclein neurotoxicity. PLoS Genet. 2023, May 18;19(5):e1010760. 10.1371/journal.pgen.1010760 PMC10231792 37200393

[37] Onur TS, Laitman A, Zhao H, Keyho R, Kim H, Wang J, et al. Downregulation of glial genes involved in synaptic function mitigates huntington’s disease pathogenesis. Elife. 2021, Apr 19;10:e64564. 10.7554/eLife.64564 PMC8149125 33871358

[38] Bates D, Mächler M, Bolker BM, Walker SC. Fitting linear mixed-effects models using lme4. J Stat Softw. 2015, Oct 7;67(1):1–48.

[39] Bowman AW. Smoothing spline ANOVA models. Biometrics. 2003, Mar 1;59(1):201–2.

[40] Wang S, Tan KL, Agosto MA, Xiong B, Yamamoto S, Sandoval H, et al. The Retromer complex is required for rhodopsin recycling and its loss leads to photoreceptor degeneration. PLoS Biol. 2014;12(4):e1001847. 10.1371/journal.pbio.1001847 PMC4004542 24781186

[41] Jung JH, Yang DQ, Song H, Wang X, Wu X, Kim KP, et al. Characterization of lipid alterations by oncogenic PIK3CA mutations using untargeted lipidomics in breast cancer. Omi A J Integr Biol. 2023, Jul 1;27(7):327–35. 10.1089/omi.2023.0076 PMC10366275 37463468

[42] Dutta D, Kanca O, Byeon SK, Marcogliese PC, Zuo Z, Shridharan R V, et al. A defect in mitochondrial fatty acid synthesis impairs iron metabolism and causes elevated ceramide levels. Nat Metab. 2023, Aug 31;5(9):1595–614. 10.1038/s42255-023-00873-0 PMC11151872 37653044

[43] Byeon SK, Madugundu AK, Pandey A. Automated data-driven mass spectrometry for improved analysis of lipids with dual dissociation techniques. J Mass Spectrom Adv Clin Lab. 2021, Nov 1;22:43–49. 10.1016/j.jmsacl.2021.10.003 PMC8662330 34939054

[44] Tan AH, Noyce A, Carrasco AM, Brice A, Reimer A, Illarionova A, et al. GP2: the global Parkinson’s Genetics Program. Mov Disord. 2021, Apr 1;36(4):842–51. 10.1002/mds.28494 PMC9290711 33513272

[45] Vitale D, Koretsky MJ, Kuznetsov N, Hong S, Martin J, James M, et al. GenoTools: an open-source python package for efficient genotype data quality control and analysis. G3. 2025, Jan 1;15(1):jkae268. 10.1093/g3journal/jkae268 PMC11708233 39566101

[46] Towns C, Richer M, Jasaityte S, Stafford EJ, Joubert J, Antar T, et al. Defining the causes of sporadic Parkinson’s disease in the global Parkinson’s genetics program (GP2). NPJ Park Dis. 2023, Sep 12;9(1):1–6. 10.1038/s41531-023-00533-w PMC10497609 37699923

[47] Lange LM, Avenali M, Ellis M, Illarionova A, Keller Sarmiento IJ, Tan AH, et al. Elucidating causative gene variants in hereditary Parkinson’s disease in the global Parkinson’s Genetics Program (GP2). npj Park Dis. 2023, Dec 1;9(1):1–5. 10.1038/s41531-023-00526-9 PMC10300084 37369645

[48] Purcell S, Neale B, Todd-Brown K, Thomas L, Ferreira MAR, Bender D, et al. Plink: a tool set for whole-genome association and population-based linkage analyses. Am J Hum Genet. 2007, Sep 1;81(3):559–75. 10.1086/519795 PMC1950838 17701901

[49] Chang CC, Chow CC, Tellier LCAM, Vattikuti S, Purcell SM, Plink LJS-G. Rising to the challenge of larger and richer datasets. Gigascience. 2015, Feb 25;4(1):7. 10.1186/s13742-015-0047-8 PMC4342193 25722852

[50] Yang H, Wang K. Genomic variant annotation and prioritization with ANNOVAR and wANNOVAR. Nat Protoc. 2015, Sep 17;10(10):1556–66. 10.1038/nprot.2015.105 PMC4718734 26379229

[51] Wang K, Li M, Hakonarson H. ANNOVAR: functional annotation of genetic variants from high-throughput sequencing data. Nucleic Acids Res. 2010, Sep 1;38(16):e164–164. 10.1093/nar/gkq603 PMC2938201 20601685

[52] Toffoli M, Chen X, Sedlazeck FJ, Lee CY, Mullin S, Higgins A, et al. Comprehensive short and long read sequencing analysis for the Gaucher and Parkinson’s disease-associated GBA gene. Commun Biol. 2022, Jul 6;5(1):1–10. 10.1038/s42003-022-03610-7 PMC9259685 35794204

[53] Burren OS, Dhindsa RS, Deevi SVV, Wen S, Nag A, Mitchell J, et al. Genetic architecture of telomere length in 462,666 UK Biobank whole-genome sequences. Nat Genet. 2024, Aug 27;56(9):1832–40. 10.1038/s41588-024-01884-7 PMC11387196 39192095

[54] Li S, Carss KJ, Halldorsson BV, Cortes A, Consortium UBW-GS. Whole-genome sequencing of half-a-million UK Biobank participants. medRxiv. 2023, Dec 8;2023.12.06.23299426.

[55] McLaren W, Gil L, Hunt SE, Riat HS, Ritchie GRS, Thormann A, et al. The ensembl variant effect predictor. Genome Biol. 2016, Jun 6;17(1):122. 10.1186/s13059-016-0974-4 PMC4893825 27268795

[56] Öztürk-Çolak A, Marygold SJ, Antonazzo G, Attrill H, Goutte-Gattat D, Jenkins VK, et al. FlyBase: updates to the Drosophila genes and genomes database. Genetics. 2024, May 7;227(1). 10.1093/genetics/iyad211 PMC11075543 38301657

[57] Jenkins VK, Larkin A, Thurmond J. Using FlyBase: a database of Drosophila genes and Genetics. In: Methods in molecular biology. Humana Press Inc; 2022. p. 1–34. 10.1007/978-1-0716-2541-5_1 35980571

[58] Kinghorn KJ, Grönke S, Castillo-Quan JI, Woodling NS, Li L, Sirka E, et al. A Drosophila model of neuronopathic Gaucher disease demonstrates lysosomal-autophagic defects and altered mTOR signalling and is functionally rescued by rapamycin. J Neurosci. 2016, Nov, 16;36(46):11654–70. 10.1523/JNEUROSCI.4527-15.2016 PMC5125225 27852774

[59] Rousseaux MWC, Vázquez-Vélez GE, Al-Ramahi I, Jeong HH, Bajić A, Revelli JP, et al. A druggable genome screen identifies modifiers of α-synuclein levels via a tiered cross-species Validation approach. J Neurosci. 2018, Oct, 24;38(43):9286–301. 10.1523/JNEUROSCI.0254-18.2018 PMC6199406 30249792

[60] van Veen S, Martin S, den Haute C V, Benoy V, Lyons J, Vanhoutte R, et al. ATP13A2 deficiency disrupts lysosomal polyamine export. Nature. 2020, Jan, 29;578(7795):419–24. 10.1038/s41586-020-1968-7 31996848

[61] Fujii T, Nagamori S, Wiriyasermkul P, Zheng S, Yago A, Shimizu T, et al. Parkinson’s disease-associated ATP13A2/PARK9 functions as a lysosomal H+, K±ATPase. Nat Commun. 2023, Apr 20;14(1):1–11. 10.1038/s41467-023-37815-z PMC10119128 37080960

[62] Ramirez A, Heimbach A, Gründemann J, Stiller B, Hampshire D, Cid LP, et al. Hereditary parkinsonism with dementia is caused by mutations in ATP13A2, encoding a lysosomal type 5 P-type ATPase. Nat Genet. 2006, Oct 10;38(10):1184–91. 10.1038/ng1884 16964263

[63] Usenovic M, Knight AL, Ray A, Wong V, Brown KR, Caldwell GA, et al. Identification of novel ATP13A2 interactors and their role in α-synuclein misfolding and toxicity. Hum Mol Genet. 2012, Sep 1;21(17):3785–94. 10.1093/hmg/dds206 PMC3412378 22645275

[64] Si J, Van den Haute C, Lobbestael E, Martin S, van Veen S, Vangheluwe P, et al. ATP13A2 regulates cellular α-synuclein multimerization, membrane association, and externalization. Int J Mol Sci. 2021, Mar 7;22(5):1–20. 10.3390/ijms22052689 PMC7962109 33799982

[65] Vrijsen S, Besora-Casals L, Van Veen S, Zielich J, Van Den Haute C, Hamouda NN, et al. ATP13A2-mediated endo-lysosomal polyamine export counters mitochondrial oxidative stress. Proc Natl Acad Sci U S A. 2020, Dec 8;117(49):31198–207. 10.1073/pnas.1922342117 PMC7733819 33229544

[66] Jumper J, Evans R, Pritzel A, Green T, Figurnov M, Ronneberger O, et al. Highly accurate protein structure prediction with AlphaFold. Nat. 2021 Aug; 596(7873):583–89. 10.1038/s41586-021-03819-2 PMC8371605 34265844

[67] Varadi M, Anyango S, Deshpande M, Nair S, Natassia C, Yordanova G, et al. AlphaFold protein structure database: massively expanding the structural coverage of protein-sequence space with high-accuracy models. Nucleic Acids Res. 2022, Jan 7;50(D1):D439–44. 10.1093/nar/gkab1061 PMC8728224 34791371

[68] Stinchfield MJ, Weasner BP, Weasner BM, Zhitomersky D, Kumar JP, O’Connor MB, et al. Fourth chromosome Resource project: a comprehensive resource for genetic analysis in Drosophila that includes humanized stocks. Genetics. 2024, Feb 7;226(2). 10.1093/genetics/iyad201 PMC10847715 37981656

[69] Ryder E, Ashburner M, Bautista-Llacer R, Drummond J, Webster J, Johnson G, et al. The DrosDel deletion collection: a Drosophila genomewide chromosomal deficiency Resource. Genetics. 2007, Sep 1;177(1):615–29. 10.1534/genetics.107.076216 PMC2013729 17720900

[70] Ravenscroft TA, Jacobs A, Gu M, Eberl DF, Bellen HJ. The voltage-Gated Sodium channel in Drosophila, para, localizes to dendrites as well as axons in mechanosensitive chordotonal neurons. eNeuro. 2023, Jun 1;10(6). 10.1523/ENEURO.0105-23.2023 PMC10316460 37328295

[71] Wherrett JR, Huterer S. Enrichment of bis-(monoacylglyceryl) phosphate in lysosomes from rat liver. J Biol Chem. 1972;247(13):4114–20. 4338481

[72] Settembre C, Zoncu R, Medina DL, Vetrini F, Erdin S, Erdin S, et al. A lysosome-to-nucleus signalling mechanism senses and regulates the lysosome via mTOR and TFEB. Embo. 2012, Mar 7;31(5):1095–108. 10.1038/emboj.2012.32 PMC3298007 22343943

[73] Shen D, Wang X, Li X, Zhang X, Yao Z, Dibble S, et al. Lipid storage disorders block lysosomal trafficking by inhibiting a trp channel and lysosomal calcium release. Nat Commun. 2012, Mar 13;3(1):1–11. 10.1038/ncomms1735 PMC3347486 22415822

[74] Stewart TM, Foley JR, Holbert CE, Khomutov M, Rastkari N, Tao X, et al. Difluoromethylornithine rebalances aberrant polyamine ratios in snyder-robinson syndrome. EMBO Mol Med. 2023, Nov 8;15(11):e17833. 10.15252/emmm.202317833 PMC10630878 37702369

[75] Bey P, Danzin C, Van DV, Mamont P, Jung M, Tardif C. Analogs of ornithine as inhibitors of ornithine decarboxylase. New deductions concerning the topography of the enzyme’s active site. J Med Chem. 2002;21(1):50–55. 10.1021/jm00199a009 619149

[76] Auvinen M, Paasinen A, Andersson LC, Hölttä E. Ornithine decarboxylase activity is critical for cell transformation. Nat. 1992, Nov 26;360(6402):355–8. 10.1038/360355a0 1280331

[77] Mallett V, Ross JP, Alcalay RN, Ambalavanan A, Sidransky E, Dion PA, et al. GBA P.T369M substitution in Parkinson disease: polymorphism or association? A meta-analysis. Neurol Genet. 2016;2(5):e104. 10.1212/NXG.0000000000000104 PMC5017539 27648471

[78] Greuel A, Trezzi JP, Glaab E, Ruppert MC, Maier F, Jäger C, et al. GBA variants in Parkinson’s disease: clinical, metabolomic, and multimodal neuroimaging phenotypes. Mov Disord. 2020, Dec 1;35(12):2201–10. 10.1002/mds.28225 32853481

[79] Benitez BA, Davis AA, Jin SC, Ibanez L, Ortega-Cubero S, Pastor P, et al. Resequencing analysis of five Mendelian genes and the top genes from genome-wide association studies in Parkinson’s disease. Mol Neurodegener. 2016, Apr 19;11(1):1–12. 10.1186/s13024-016-0097-0 PMC4837564 27094865

[80] Parlar SC, Grenn FP, Kim JJ, Baluwendraat C, Gan-Or Z. Classification of GBA1 variants in Parkinson’s disease: the GBA1-PD browser. Mov Disord. 2023, Mar 1;38(3):489–95. 10.1002/mds.29314 PMC10033371 36598340

[81] Wang J, Al-Ouran R, Hu Y, Kim SY, Wan YW, Wangler MF, et al. MARRVEL: integration of human and model organism genetic Resources to facilitate functional annotation of the human genome. Am J Hum Genet. 2017, Jun 1;100(6):843–53. 10.1016/j.ajhg.2017.04.010 PMC5670038 28502612

[82] Boddupalli CS, Nair S, Belinsky G, Gans J, Teeple E, Nguyen TH, et al. Neuroinflammation in neuronopathic Gaucher disease: role of microglia and NK cells, biomarkers, and response to substrate reduction therapy. Elife. 2022, Aug 1;11. 10.7554/eLife.79830 PMC9381039 35972072

[83] Keatinge M, Bui H, Menke A, Chen YC, Sokol AM, Bai Q, et al. Glucocerebrosidase 1 deficient danio rerio mirror key pathological aspects of human Gaucher disease and provide evidence of early microglial activation preceding alpha-synuclein-independent neuronal cell death. Hum Mol Genet. 2015, Dec 1;24(23):6640–52. 10.1093/hmg/ddv369 PMC4634372 26376862

[84] Brunialti E, Villa A, Szego EM, La Vitola P, Drago D, Pavlovic R, et al. Metabolic reprogramming and altered ATP content impair neuroprotective functions of microglia in β-glucocerebrosidase deficiency models. J Neuroinflammation. 2025, 221. 2025 Nov 25;22(1):279. 10.1186/s12974-025-03616-y PMC12648879 41291747

[85] Blumenreich S, Nehushtan T, Kupervaser M, Shalit T, Gabashvili A, Joseph T, et al. Large-scale proteomics analysis of five brain regions from Parkinson’s disease patients with a GBA1 mutation. npj Park Dis. 2024, Dec 1;10(1):1–15. 10.1038/s41531-024-00645-x PMC10853186 38331996

[86] Troshev D, Blokhin V, Ukrainskaya V, Kolacheva A, Ugrumov M. Isolation of living dopaminergic neurons labeled with a fluorescent ligand of the dopamine transporter from mouse substantia Nigra as a new tool for basic and applied research. Front Mol Neurosci. 2022, Dec 9;15:1020070. 10.3389/fnmol.2022.1020070 PMC9780273 36568278

[87] Heikkila RE, Manzino L, Cabbat FS, Duvoisin RC. Protection against the dopaminergic neurotoxicity of 1-methyl-4-phenyl-1, 2, 5, 6-tetrahydropyridine by monoamine oxidase inhibitors. Nature. 1984;311(5985):467–69. 10.1038/311467a0 6332989

[88] Guedes LC, Chan RB, Gomes MA, Conceição VA, Machado RB, Soares T, et al. Serum lipid alterations in GBA-associated Parkinson’s disease. Park Relat Disord. 2017, Nov 1;44:58–65. 10.1016/j.parkreldis.2017.08.026 28890071

[89] Abbott SK, Li H, Muñoz SS, Knoch B, Batterham M, Murphy KE, et al. Altered ceramide acyl chain length and ceramide synthase gene expression in Parkinson’s disease. Mov Disord. 2014, Apr 1;29(4):518–26. 10.1002/mds.25729 24822250

[90] Fernández-Irigoyen J, Cartas-Cejudo P, Iruarrizaga-Lejarreta M, Santamaría E. Alteration in the cerebrospinal fluid lipidome in Parkinson’s disease: a post-mortem pilot study. Biomedicines. 2021, May 1;9(5):491. 10.3390/biomedicines9050491 PMC8146703 33946950

[91] Xing Y, Tang Y, Zhao L, Wang Q, Qin W, Ji X, et al. Associations between plasma ceramides and cognitive and neuropsychiatric manifestations in Parkinson’s disease dementia. J Neurol Sci. 2016, Nov 15;370:82–87. 10.1016/j.jns.2016.09.028 27772793

[92] Mielke MM, Maetzler W, Haughey NJ, Bandaru VVR, Savica R, Deuschle C, et al. Plasma ceramide and glucosylceramide metabolism is altered in sporadic Parkinson’s disease and associated with cognitive impairment: a pilot study. PLoS One. 2013, Sep 18;8(9):e73094. 10.1371/journal.pone.0073094 PMC3776817 24058461

[93] Cheng D, Jenner AM, Shui G, Cheong WF, Mitchell TW, Nealon JR, et al. Lipid pathway alterations in Parkinson’s disease primary visual cortex. PLoS ONE. 2011;6(2):e17299. 10.1371/journal.pone.0017299 PMC3046155 21387008

[94] Blumenreich S, Nehushtan T, Barav OB, Saville JT, Dingjan T, Hardy J, et al. Elevation of gangliosides in four brain regions from Parkinson’s disease patients with a GBA mutation. NPJ Park Dis. 2022, Dec 1;8(1):1–11. 10.1038/s41531-022-00363-2 PMC9357011 35933559

[95] Lin G, Lee PT, Chen K, Mao D, Tan KL, Zuo Z, et al. Phospholipase PLA2G6, a parkinsonism-associated gene, Affects Vps26 and Vps35, Retromer function, and ceramide levels, similar to α-synuclein gain. Cell Metab. 2018, Oct 2;28(4):605–18.e6. 10.1016/j.cmet.2018.05.019 29909971

[96] Vos M, Dulovic-Mahlow M, Mandik F, Frese L, Kanana Y, Diaw SH, et al. Ceramide accumulation induces mitophagy and impairs β-oxidation in PINK1 deficiency. Proc Natl Acad Sci. U S A. 2021, Oct 26;118(43):e2025347118. 10.1073/pnas.2025347118 PMC8639384 34686591

[97] Pan X, Dutta D, Lu S, Bellen HJ. Sphingolipids in neurodegenerative diseases. Front Neurosci. 2023, Feb 16;17:1137893. 10.3389/fnins.2023.1137893 PMC9978793 36875645

[98] Rayaprolu S, Seven YB, Howard J, Duffy C, Altshuler M, Moloney C, et al. Partial loss of ATP13A2 causes selective gliosis independent of robust lipofuscinosis. Mol Cell Neurosc. 2018, Oct 1;92:17–26. 10.1016/j.mcn.2018.05.009 29859891

[99] Kaempf N, Valadas JS, Robberechts P, Schoovaerts N, Praschberger R, Ortega A, et al. Behavioral screening defines three molecular parkinsonism subgroups in Drosophila. bioRxiv. 2024, Aug 27;2024.08.27.609924. 10.1038/s41467-026-70303-8 PMC13106710 41807392

[100] Dhanushkodi NR, Abul Khair SB, Ardah MT, Haque ME. ATP13A2 gene silencing in Drosophila Affects autophagic degradation of A53T mutant α-synuclein. Int J Mol Sci. 2023, Jan 16;24(2):1775. 10.3390/ijms24021775 PMC9861907 36675288

[101] Vrijsen S, Houdou M, Cascalho A, Eggermont J, Vangheluwe P. Polyamines in Parkinson’s disease: balancing between neurotoxicity and Neuroprotection. Annu Rev Biochem. 2023, Jun 20;2023:435–64. 10.1146/annurev-biochem-071322-021330 37018845

[102] Settembre C, Di Malta C, Polito VA, Arencibia MG, Vetrini F, Erdin S, et al. TFEB links autophagy to lysosomal biogenesis. Sci. 2011, Jun 17;332(6036):1429–33. 10.1126/science.1204592 PMC3638014 21617040

[103] Awad O, Sarkar C, Panicker LM, Miller D, Zeng X, Sgambato JA, et al. Altered TFEB-mediated lysosomal biogenesis in Gaucher disease iPSC-derived neuronal cells. Hum Mol Genet. 2015, Oct 15;24(20):5775–88. 10.1093/hmg/ddv297 26220978

[104] Pintavorn P, Munie S, Munagapati S. Lamellar bodies in podocytes associated with compound heterozygous mutations for Niemann Pick type C1 mimicking Fabry disease, a Case report. Can J Kidney Heal Dis. 2022, Oct 28;9:20543581221124635. 10.1177/20543581221124635 PMC9619285 36325261

[105] Owens CL, Russell SD, Halushka MK. Histologic and electron microscopy findings in myocardium of treated Fabry disease. Hum Pathol. 2006, Jun 1;37(6):764–68. 10.1016/j.humpath.2006.01.021 16733219

[106] Li X, Xiang C, Zhu S, Guo J, Liu C, Wang A, et al. SNX8 enables lysosome reformation and reverses lysosomal storage disorder. Nat Commun. 2024, Mar 22;15(1):1–15. 10.1038/s41467-024-46705-x PMC10959956 38519472

[107] Coste H, Martel MB, Got R. Topology of glucosylceramide synthesis in Golgi membranes from porcine submaxillary glands. BBA - Biomembr. 1986, Jun 13;858(1):6–12. 10.1016/0005-2736(86)90285-3 2939881

[108] Jeckel D, Karrenbauer A, Burger KNJ, Van Meer G, Wieland F. Glucosylceramide is synthesized at the cytosolic surface of various Golgi subfractions. J Cell Biol. 1992, Apr 15;117(2):259–67. 10.1083/jcb.117.2.259 PMC2289419 1532799

[109] Brenkert A, Radin NS. Synthesis of galactosyl ceramide and glucosyl ceramide by rat brain: assay procedures and changes with age. Brain Res. 1972, Jan 14;36(1):183–93. 10.1016/0006-8993(72)90774-3 4257605

[110] Radin NS, Brenkert A, Arora RC, Sellinger OZ, Flangas AL. Glial and neuronal localization of cerebroside-metabolizing enzymes. Brain Res. 1972, Apr 14;39(1):163–69. 10.1016/0006-8993(72)90792-5 5025640

[111] D’Angelo G, Polishchuk E, Di TG, Santoro M, Di CA, Godi A, et al. Glycosphingolipid synthesis requires FAPP2 transfer of glucosylceramide. Nature. 2007, Sep 6;449(7158):62–67. 10.1038/nature06097 17687330

[112] Martin OC, Pagano RE. Internalization and sorting of a fluorescent analogue of glucosylceramide to the Golgi apparatus of human skin fibroblasts: utilization of endocytic and nonendocytic transport mechanisms. J Cell Biol. 1994, May 15;125(4):769–81. 10.1083/jcb.125.4.769 PMC2120081 8188745

[113] Lipsky NG, Pagano RE. Intracellular translocation of fluorescent sphingolipids in cultured fibroblasts: endogenously synthesized sphingomyelin and glucocerebroside analogues pass through the Golgi apparatus en route to the plasma membrane. J Cell Biol. 1985, Jan 1;100(1):27–34. 10.1083/jcb.100.1.27 PMC2113465 3965473

[114] Bähner M, Frechter S, Da Silva N, Minke B, Paulsen R, Huber A. Light-regulated subcellular translocation of drosophila TRPL channels induces long-term adaptation and modifies the light-induced current. Neuron. 2002, Mar 28;34(1):83–93. 10.1016/s0896-6273(02)00630-x 11931743

[115] Kosloff M, Elia N, Joel-Almagor T, Timberg R, Zars TD, Hyde DR, et al. Regulation of light-dependent Gqα translocation and morphological changes in fly photoreceptors. Embo J. 2003, Feb 3;22(3):459–68. 10.1093/emboj/cdg054 PMC140738 12554647

[116] Han J, Reddig K, Li HS. Prolonged Gq activity triggers fly rhodopsin endocytosis and degradation, and reduces photoreceptor sensitivity. Embo J. 2007, Dec 12;26(24):4966–73. 10.1038/sj.emboj.7601929 PMC2140116 18034157

[117] Stark WS, Schilly D, Christianson JS, Bone RA, Landrum JT. Photoreceptor-specific efficiencies of β-carotene, zeaxanthin and lutein for photopigment formation deduced from receptor mutant Drosophila melanogaster. J Comp Physiol A Sens, Neural Behav Physiol. 1990 Feb;166(4):429–36. 10.1007/BF00192014 2110249

[118] Minakaki G, Safren N, Bustos BI, Lubbe SJ, Mencacci NE, Krainc D. Commander complex regulates lysosomal function and is implicated in Parkinson’s disease risk. Sci. 2025, Apr 11;388(6743):204–11. 10.1126/science.adq6650 40209002

[119] Shelke GV, Williamson CD, Jarnik M, Bonifacino JS. Inhibition of endolysosome fusion increases exosome secretion. J Cell Biol. 2023, Jun 5;222(6). 10.1083/jcb.202209084 PMC10202829 37213076

[120] Falguières T, Luyet PP, Bissig C, Scott CC, Velluz MC, Gruenberg J. In vitro budding of intralumenal vesicles into late endosomes is regulated by alix and tsg101. Mol Biol Cell. 2008, Nov 3;19(11):4942–55. 10.1091/mbc.E08-03-0239 PMC2575168 18768755

[121] Choezom D, Gross JC. Neutral sphingomyelinase 2 controls exosome secretion by counteracting V-ATPase-mediated endosome acidification. J Cell Sci. 2022, Mar 1;135(5). 10.1242/jcs.259324 PMC8919340 35050379

[122] Kobayashi T, Stang E, Fang KS, De Moerloose P, Parton RG, Gruenberg J. A lipid associated with the antiphospholipid syndrome regulates endosome structure and function. Nature. 1998, Mar 12;392(6672):193–97. 10.1038/32440 9515966

[123] Kobayashi T, Beuchat MH, Chevallier J, Makino A, Mayran N, Escola JM, et al. Separation and characterization of late endosomal membrane domains. J Biol Chem. 2002, Aug, 30;277(35):32157–64. 10.1074/jbc.M202838200 12065580

[124] Davis MY, Trinh K, Thomas RE, Yu S, Germanos AA, Whitley BN, et al. Glucocerebrosidase deficiency in Drosophila results in α-synuclein-independent protein aggregation and neurodegeneration. PLoS Genet. 2016, Mar 1;12(3):e1005944. 10.1371/journal.pgen.1005944 PMC4809718 27019408

[125] Thomas RE, Vincow ES, Merrihew GE, MacCoss MJ, Davis MY, Pallanck LJ. Glucocerebrosidase deficiency promotes protein aggregation through dysregulation of extracellular vesicles. PLoS Genet. 2018, Sep 1;14(9):e1007694. 10.1371/journal.pgen.1007694 PMC6175534 30256786

[126] Hertz E, Chen Y, Sidransky E. Gaucher disease provides a unique window into Parkinson disease pathogenesis. vol. Nat Rev Neurol. 2024;20:526–40. 10.1038/s41582-024-00999-z 39107435

[127] Straniero L, Rimoldi V, Monfrini E, Bonvegna S, Melistaccio G, Lake J, et al. Role of lysosomal gene variants in modulating GBA-Associated Parkinson’s disease risk. Mov Disord. 2022, Jun 1;37(6):1202–10. 10.1002/mds.28987 PMC9310717 35262230

[128] Bennett LL. Gaucher disease. in: frontiers in lysosomal storage diseases (LSD) treatments. 2023. StatPearls Publishing. p. 31–56.

[129] Alcalay RN, Levy OA, Waters CC, Fahn S, Ford B, Kuo SH, et al. Glucocerebrosidase activity in Parkinson’s disease with and without GBA mutations. Brain. 2015, Sep 1;138(9):2648–58. 10.1093/brain/awv179 PMC4564023 26117366

[130] Pchelina S, Baydakova G, Nikolaev M, Senkevich K, Emelyanov A, Kopytova A, et al. Blood lysosphingolipids accumulation in patients with parkinson’s disease with glucocerebrosidase 1 mutations. Mov Disord. 2018, Aug 1;33(8):1325–30. 10.1002/mds.27393 30192031

[131] Huh YE, Chiang MSR, Locascio JJ, Liao Z, Liu G, Choudhury K, et al. β-glucocerebrosidase activity in GBA-linked Parkinson disease: the type of mutation matters. Neurology. 2020, Aug 11;95(6):E685–96. 10.1212/WNL.0000000000009989 PMC7455354 32540937

[132] Lubbe SJ, Escott-Price V, Gibbs JR, Nalls MA, Bras J, Price TR, et al. Additional rare variant analysis in Parkinson’s disease cases with and without known pathogenic mutations: evidence for oligogenic inheritance. Hum Mol Genet. 2016, Dec 15;25(24):5483–89. 10.1093/hmg/ddw348 PMC5418836 27798102

