# Supplementary material for: Two lysosomal genes ATP13A2 and GBA1 interact to drive neurodegeneration
Source: Mol Neurodegener. 2026 Jan 30;21:18. doi: 10.1186/s13024-025-00923-z (PMC13001375; doi:10.1186/s13024-025-00923-z)
Supplement: Supplementary file 1 — Supplementary Material 1 [file 13024_2025_923_MOESM1_ESM.pdf]

ADDITIONAL FILE 1: SUPPLEMENTAL FIGURES, TABLES, AND REFERENCES

Two lysosomal genes *ATP13A2* and *GBA1* interact to drive neurodegeneration

Mingxue Gu, Jinghan Zhao, Mingxi Deng, Guang Lin, Xueyang Pan, Wenwen Lin, Mengqi Ma, Jinyong Kim, Seul Kee Byeon, Akhilesh Pandey, Lara Lange, Chad A. Shaw, Jonggeol Kim, Joanne Trinh, Christine Klein, Oguz Kanca, Joshua M. Shulman, and Hugo J. Bellen

Supplementary Figure Legends

Figure S1 Screening of lysosomal genes identified candidates interacting with *Gba1.* (A-I) Climbing phenotypes of flies with partial loss of indicated lysosomal genes (n=15).

Figure S2 *Gba1b-/+;anne-/+* flies show age-dependent neurodegeneration. (A) Reagent used in this study. (B) Summary of the lethality phenotype of flies lack *anne*: (i) $y^1w^*;anne^{1\text{-}205AA/1\text{-}205AA}$, (ii) $y^1w^*;anne^{T2A/1\text{-}205AA}$, (iii) $y^1w^*;anne^{1\text{-}205AA/}/Df$ (n>100). (C) Climbing analysis of the flies of the indicated genotypes after 7, 14, 21 or 30 days. $y^1w^*;Gba1b^{STOP}/+;anne^{1\text{-}205AA}/+$ flies have severe climbing defects starting from D21. Flies were raised at 25°C (n>20). (D) ERG recordings of flies of the indicated genotypes after 30 days of constant light. $y^1w^*;Gba1b^{STOP}/+;anne^{T2A}/+$ and $y^1w^*;Gba1b^{STOP}/+;anne^{1\text{-}205}/+$ flies show reduction of LCRPs and on transients. The ERG LCRP amplitudes is quantified on the right (n>6). Error bars represent SEM; **P*<0.05, ***P*<0.01, ****P*<0.001, *****P*<0.0001.

Figure S3 *anne* is predominantly expressed in active neurons in 3rd instar larvae brain. (A) Expression pattern of *anne* in the L3 larval CNS is visualized using $anne^{T2A\text{-}Gal4}$ allele-driven expression of *UAS-mCherry.NLS* (magenta) co-stained with markers for neurons (Elav) or glia (Repo) (green). Single-layer confocal images from the dashed squares indicate that mCherry is co-localized with Elav (c’) but not Repo (f’). Scale bars, 50μm and 20μm. n>3.

Figure S4 Aged $Gba1b^{T2A}/+;anne^{T2A}/+$ flies show loss of DA neurons and boutons at NMJ. (A) (a) Schematic representation of the main clusters of dopaminergic neurons in the fly brain. Adapted from Siju *et al.,* 2021(1). (b-e) Single-layer confocal images from TH staining of the PAM cluster of the indicated genotypes after 15 or 30 days of constant light. Scale bars, 20μm. (f) Quantification of TH-positive dopamine neuron counts in PAM, PPM1/2, PPM3, PPL1, PPL2, and PAL clusters (n>6). Error bars represent SEM; **P*<0.05, ***P*<0.01, ****P*<0.001, *****P*<0.0001.

Figure S5 The endo-lysosomal pathway of $Gba1b^{T2A}/+;anne^{T2A}/+$ flies is defective. (A) CTSL immunostaining (a, b) in adult brains of the indicated genotypes after 15 days of constant light. Fluorescence quantification is shown in c (n>3). Error bars represent SEM; **P*=0.034. (B) Western blots for Rab5, Dynamin, and Rab7 from adult brains of indicated genotypes after 15 days of constant light. Mild increases in Rab5, Dynamin, and Rab7 are observed in $w+; Gba1b^{T2A}/+; anne^{T2A}/+$ flies (n > 3). Quantification is shown in b-d. Error bars represent SEM; **P*=0.0403, 0.0347, 0.0106. (C) Live imaging of LysoTracker Red DN-99 in the fat body of adult flies of indicated genotypes after 15 days of constant light. $w+;Gba1b^{T2A}/+;anne^{T2A}/+$ flies show defective lysosome acidification. Scale bars, 20μm. LysoTracker puncta number and intensity are quantified in e and f. Error bars represent SEM; ****P*<0.001, *****P*<0.0001. (D) qRT-PCR analyses of V100-2, VhaSFD, Vha16-1, Vha68-2, VhaAC45, Atg8a, V100-1, and VhaM8.9 expression in adult brains of the indicated genotypes after 15 days of constant, normalized to GAPDH. n=3 biological replicates, each with 3 technical replicates. Error bars represent SEM. (E) Western blot for S6K, pS6K, Atg8 (a), and Ubiquitin (b) with protein lysates from adult brains of the indicated genotypes after 30 days of constant light. No differences were observed in the levels of the proteins in $w+;Gba1b^{T2A}/+;anne^{T2A}/+$ flies (n=3).

Figure S6 Sphingolipid and polyamine metabolism are dysregulated in *Gba1b*^{T2A}/+;*anne*^{T2A}/+ flies. (A) Principle component analysis comparing the levels of the 780 metabolites (see Additional File 3) between *w*+ heads, *w+;Gba1b*^{T2A}/+ heads, *w+;anne*^{T2A}/+ heads, *and w+;Gba1b*^{T2A}/+;*anne*^{T2A}/+ heads collected at day 7, 15, 30. (B) Fold changes in GlcCer species levels in *w+;Gba1b*^{T2A}/+;*anne*^{T2A}/+ heads compared to *w*+ at day 30 (n = 5). (C) (a) Diagram of the polyamine catabolism pathway. PUT, putrescine; SPD, spermidine; SPM, spermine; AcSPD, acetylspermidine; AcSPM, acetylspermine. (b) Heat map showing the mean $\log_2$-transformed fold changes in metabolites involved in polyamine metabolism in *w*+ and *w+;Gba1b*^{T2A}/+;*anne*^{T2A}/+ heads at days 7, 15, and 30. (c and d) Fold changes in spermidine (c) and spermine (d) levels in the indicated genotypes at days 7, 15, and 30 (n = 5).

Figure S7 Inhibiting polyamide synthesis rescues the neurodegeneration in aged *Gba1b*^{T2A}/+;*anne*^{T2A}/+ flies. (A) Climbing analysis of flies of the indicated genotypes treated with DMSO or 10mM DFMO for 30 days. DFMO significantly improves climbing performance. Error bars represent SEM; *P<0.05, ***P<0.001, ****P<0.0001. (B) ERG recordings (a, b) of flies of the indicated genotypes treated with DMSO or 10mM DFMO for 30 days in constant light. The reduction in the LCRPs and on-transients in *w+;Gba1b*^{T2A}/+;*anne*^{T2A}/+ flies were rescued with DFMO feeding. Quantification shown in c and d (n>5). Error bars represent SEM; *P<0.05, **P<0.01, ***P<0.001, ****P<0.0001.

Figure S8 GlcCer is accumulated in *Gba1b*^{T2A}/+;*anne*^{T2A}/+ fly retina. (A) BODIPY 493/503 (a-d) and glucosylceramide (e-h) staining of the retina of indicated genotypes after 30 days of constant light. The mean intensity of glucosylceramide is quantified in m (n>6). Scale bars, 5µm.

Figure S9 The neurodegenerative phenotypes in *Gba1b*$^{-}$/+;*anne*^{T2A}/+ flies are activity-dependent. (A) ERG recordings of flies of the indicated genotypes after 15, 30 or 45 days of constant dark. *w+;Gba1b*^{T2A}/+;*anne*^{T2A}/+ flies show no reduction of LCRPs and on-transient at the three time points. The ERG LCRP and on-transient amplitudes are quantified in m and n (n>6). Error bars represent SEM; *P<0.05, **P<0.01, ***P<0.001, ****P<0.0001.

Fig S1 Screening of lysosomal genes identified candidates interacting with *Gba1b*

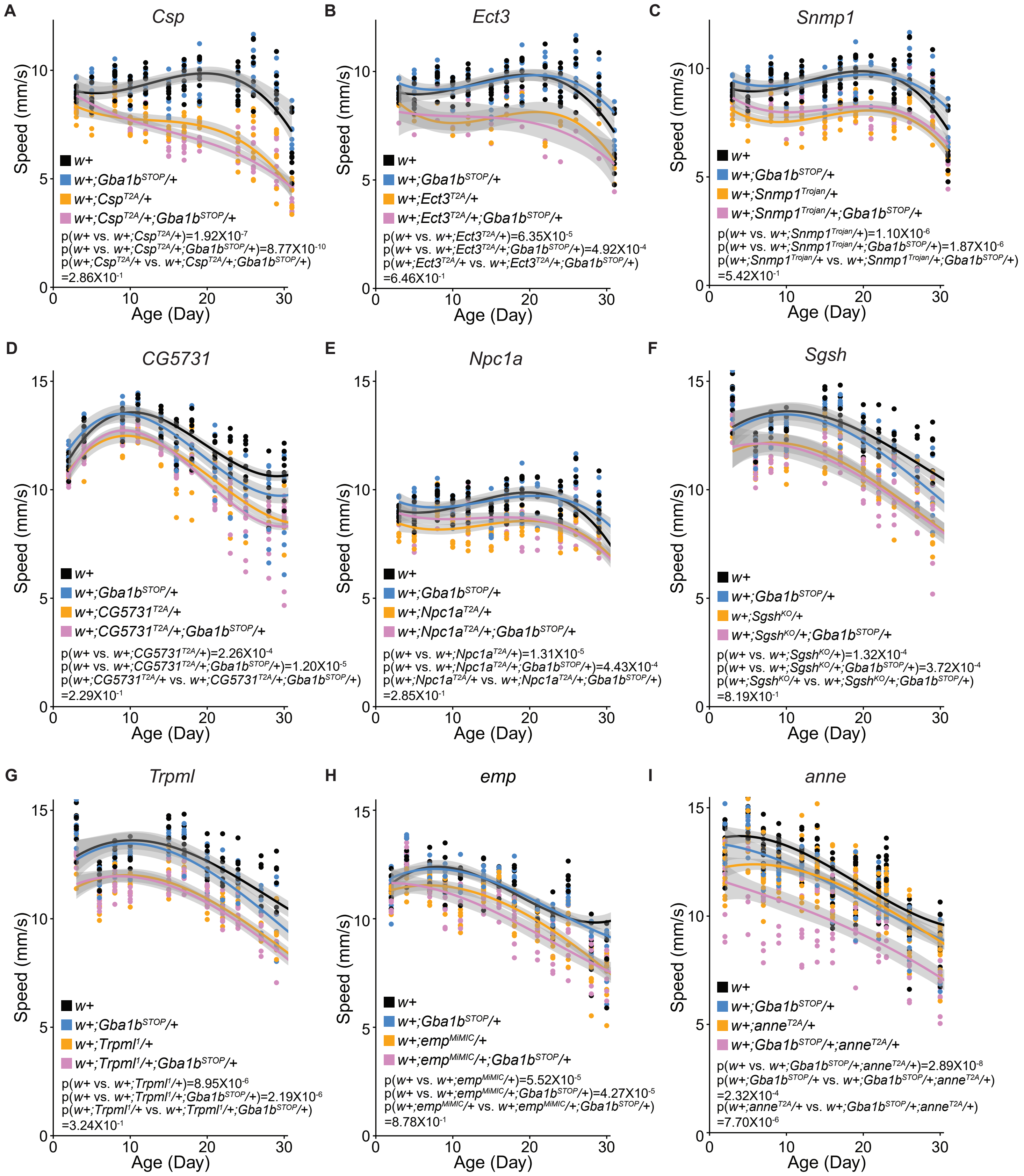

Fig S2 *Gba1b-/+;anne-/+* flies show age-dependent neurodegeneration

A Schemetic of the *anne*$^{1\text{-}205AA}$ LOF allele

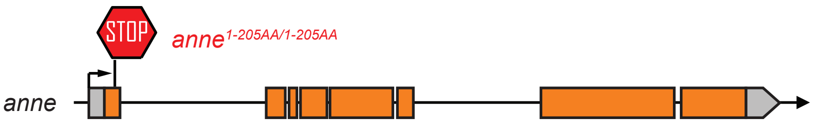

B *anne*$^{1\text{-}205AA}$ LOF is lethal

	anne$^{1\text{-}205AA}$	*anne*^{T2A}	*Df(4)ED6364*
anne$^{1\text{-}205AA}$	Embryonic Lethal	Embryonic Lethal	Embryonic Lethal

C *Gba1b-/+;anne-/+* flies show age-dependent climbing defects

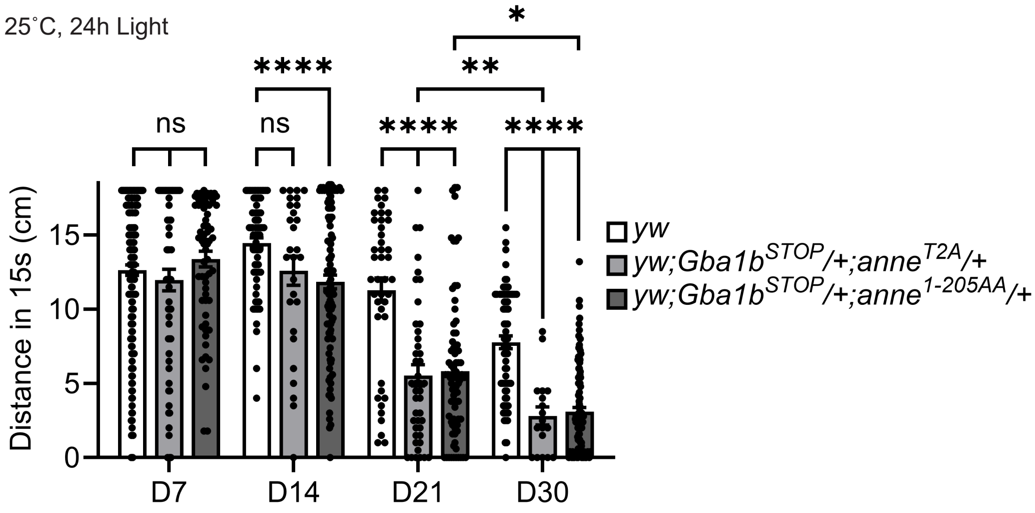

D Aged *Gba1b-/+;anne-/+* flies show ERG defects

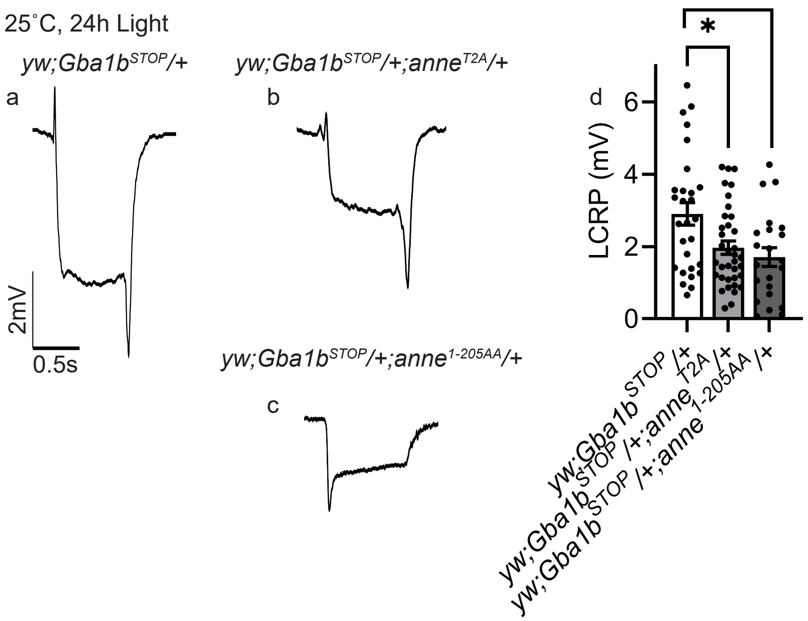

Fig S3 *anne* is predominantly expressed in active neurons in 3rd instar larvae brain

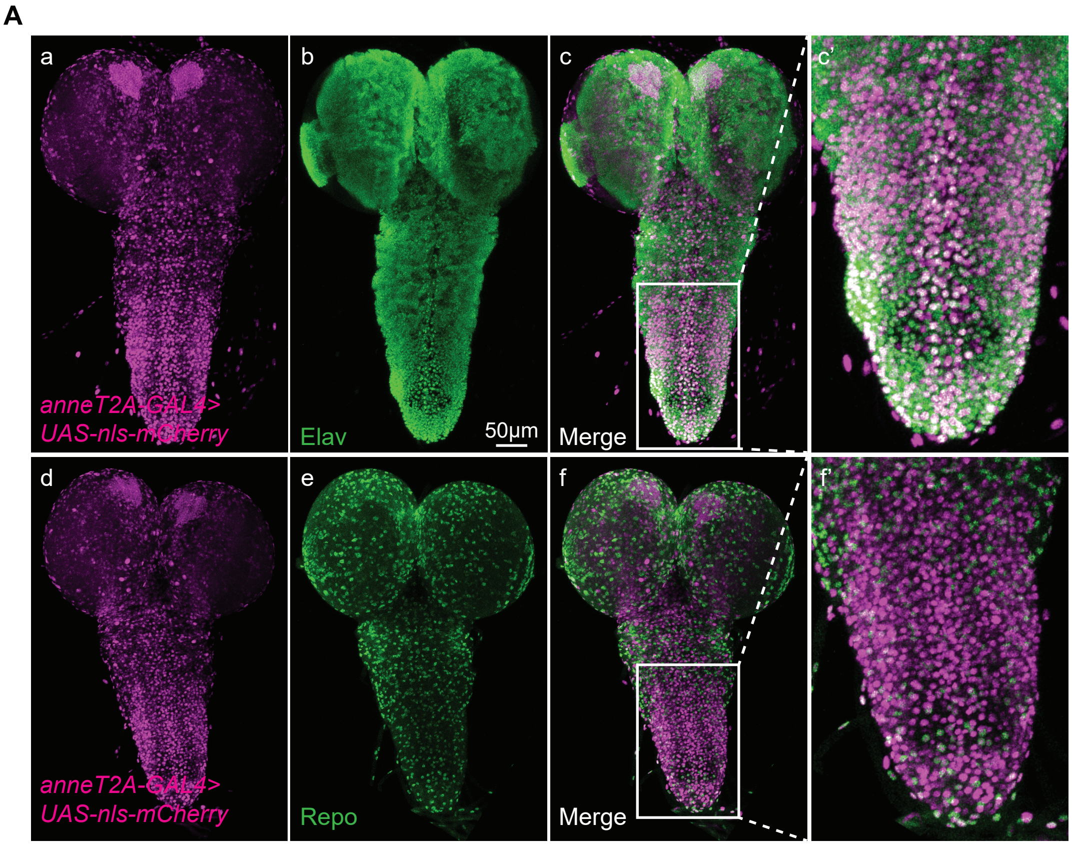

Fig S4 Aged *Gba1b*^{T2A}*/+;anne*^{T2A}*/+* flies show loss of DA neurons

A *Gba1b*^{T2A}*/+;anne*^{T2A}*/+* flies show age-dependent loss of DA neurons

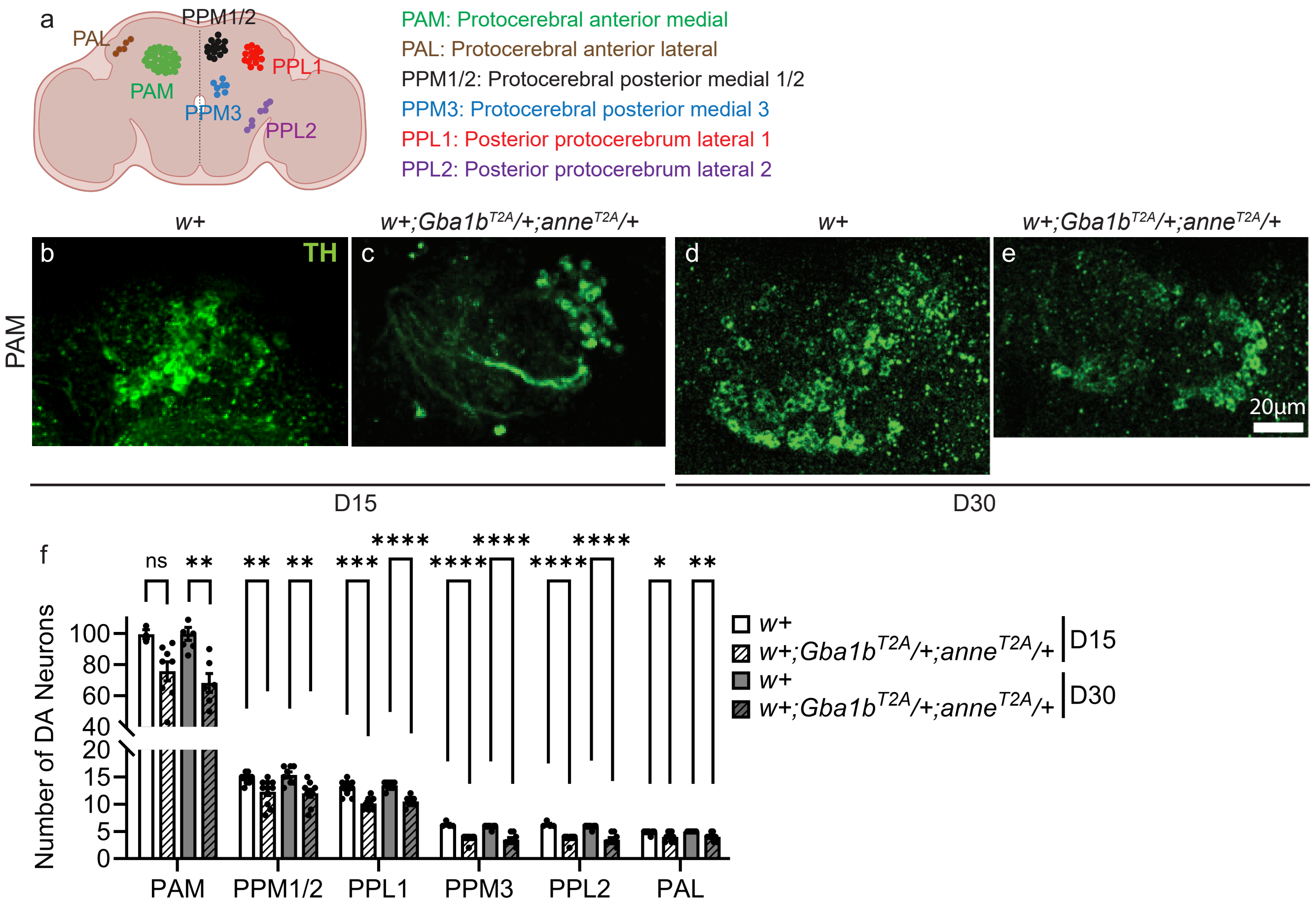

Fig S5 The endo-lysosomal pathway of *Gba1b*^{T2A}/+;*anne*^{T2A}/+ flies is defective, related to Figure 4

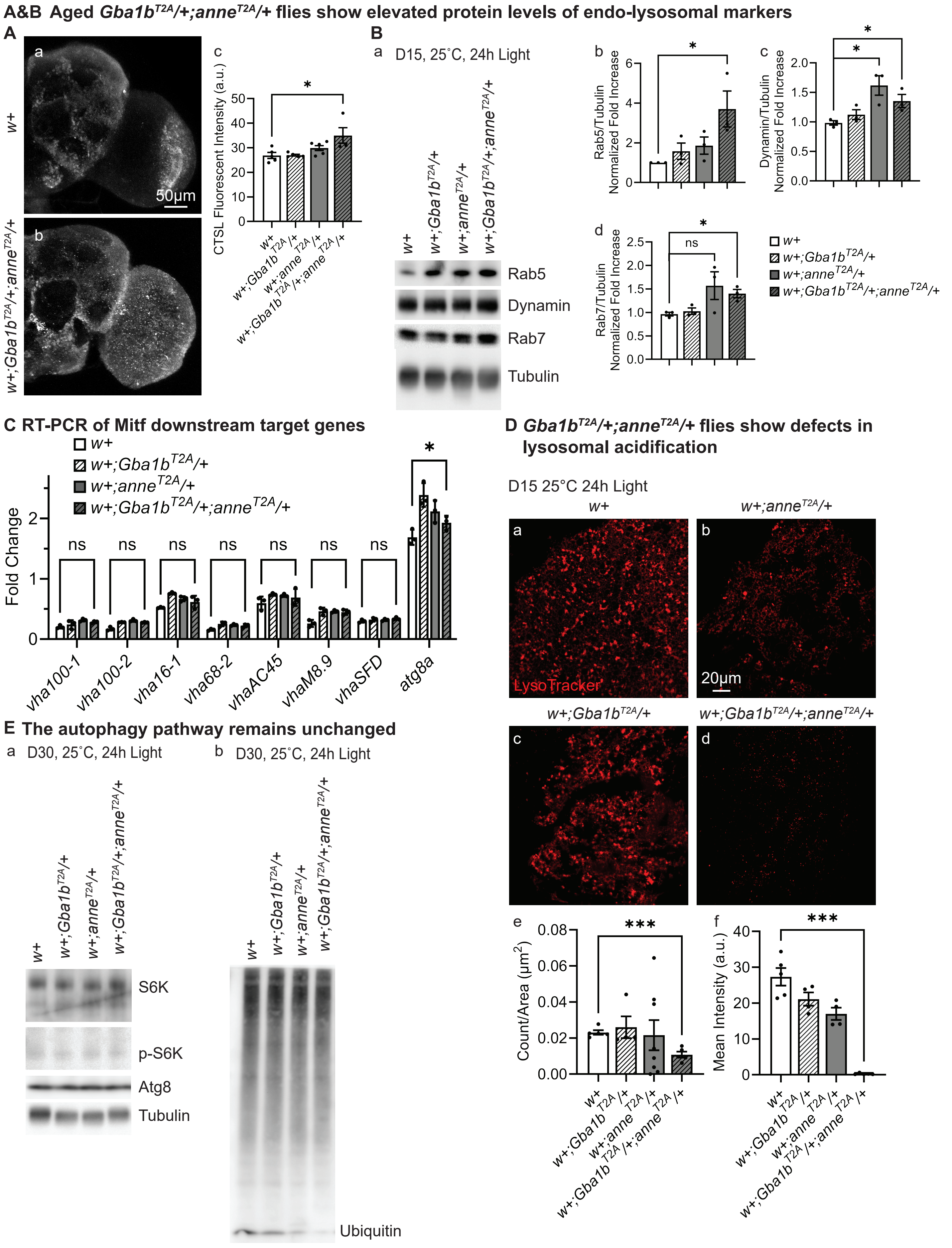

Fig S6 Sphingolipid and polyamine metabolism are dysregulated in *Gba1b*^{T2A}/+;*anne*^{T2A}/+ flies

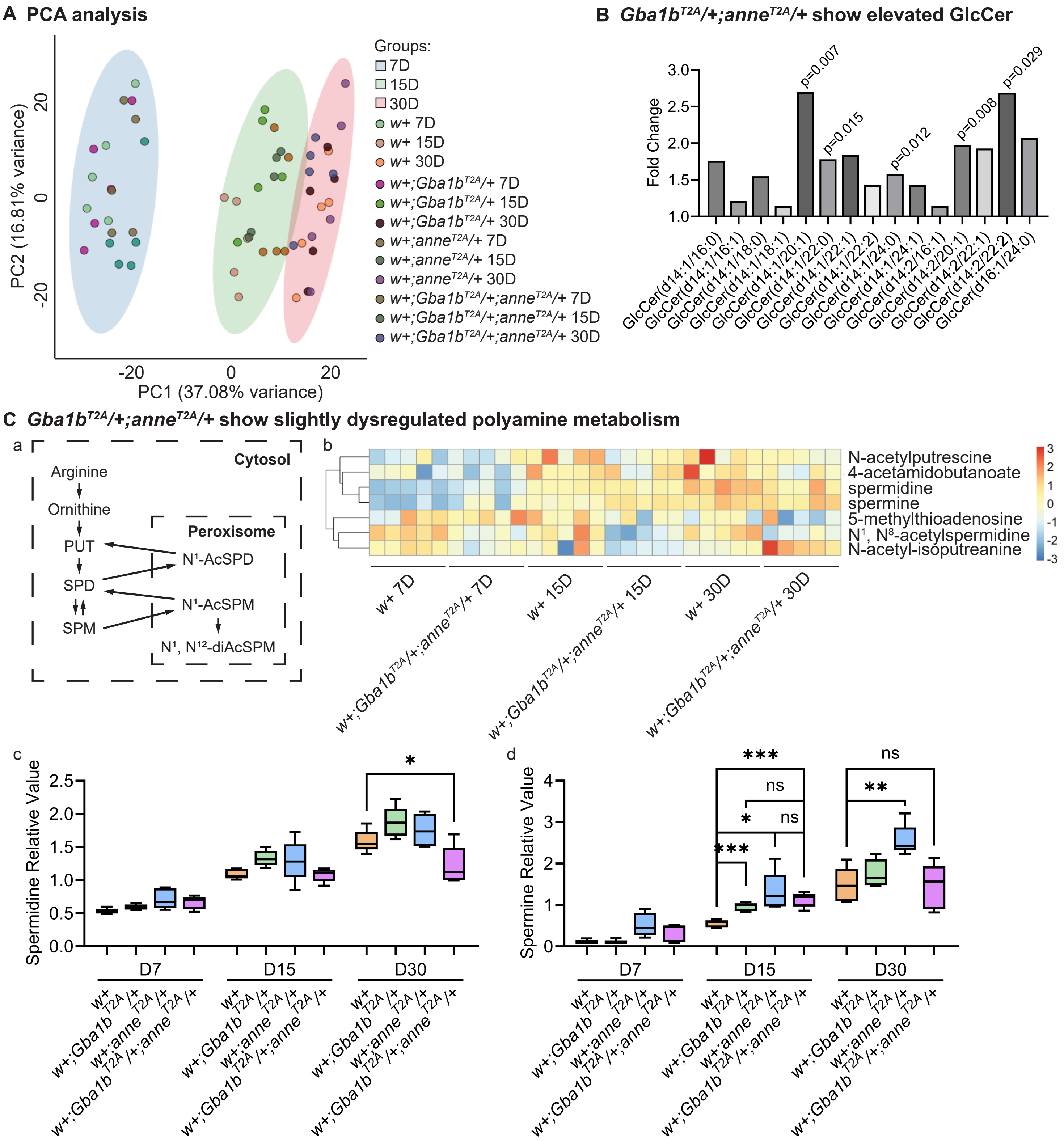

Fig S7 Inhibiting polyamide synthesis rescues the neurodegeneration in aged *Gba1b^{T2A}/+;anne^{T2A}*/+ flies

10mM DFMO partially rescued the climbing and ERG defects in aged *Gba1b^{T2A}/+;anne^{T2A}*/+ flies

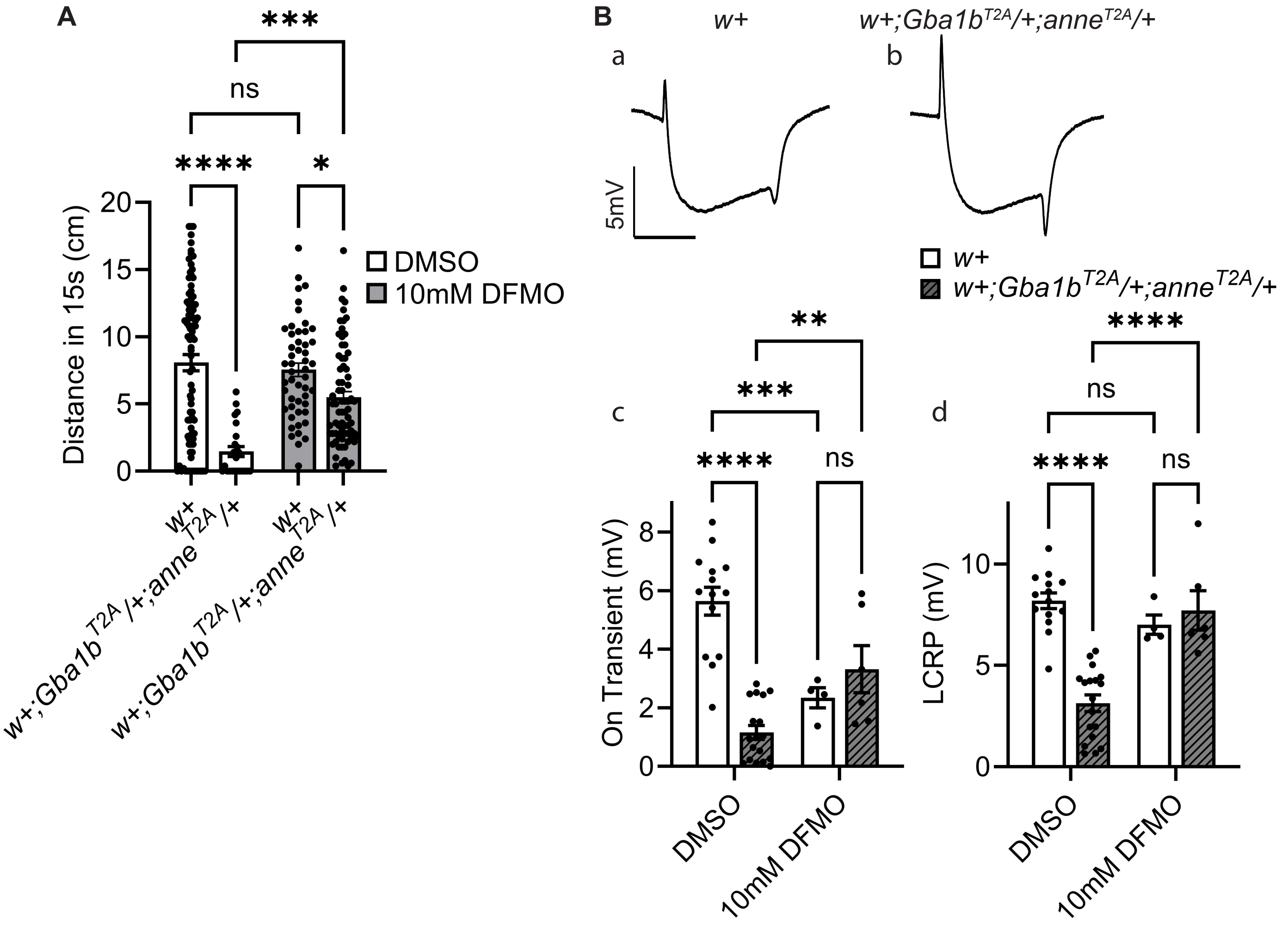

Fig S8 GlcCer is accumulated in *Gba1b*^{T2A}/+;*anne*^{T2A}/+ fly retina

A *Gba1b*^{T2A}/+;*anne*^{T2A}/+ show elevated GlcCer in pigment glia as well as photoreceptors at D30 upon neurodgeneration

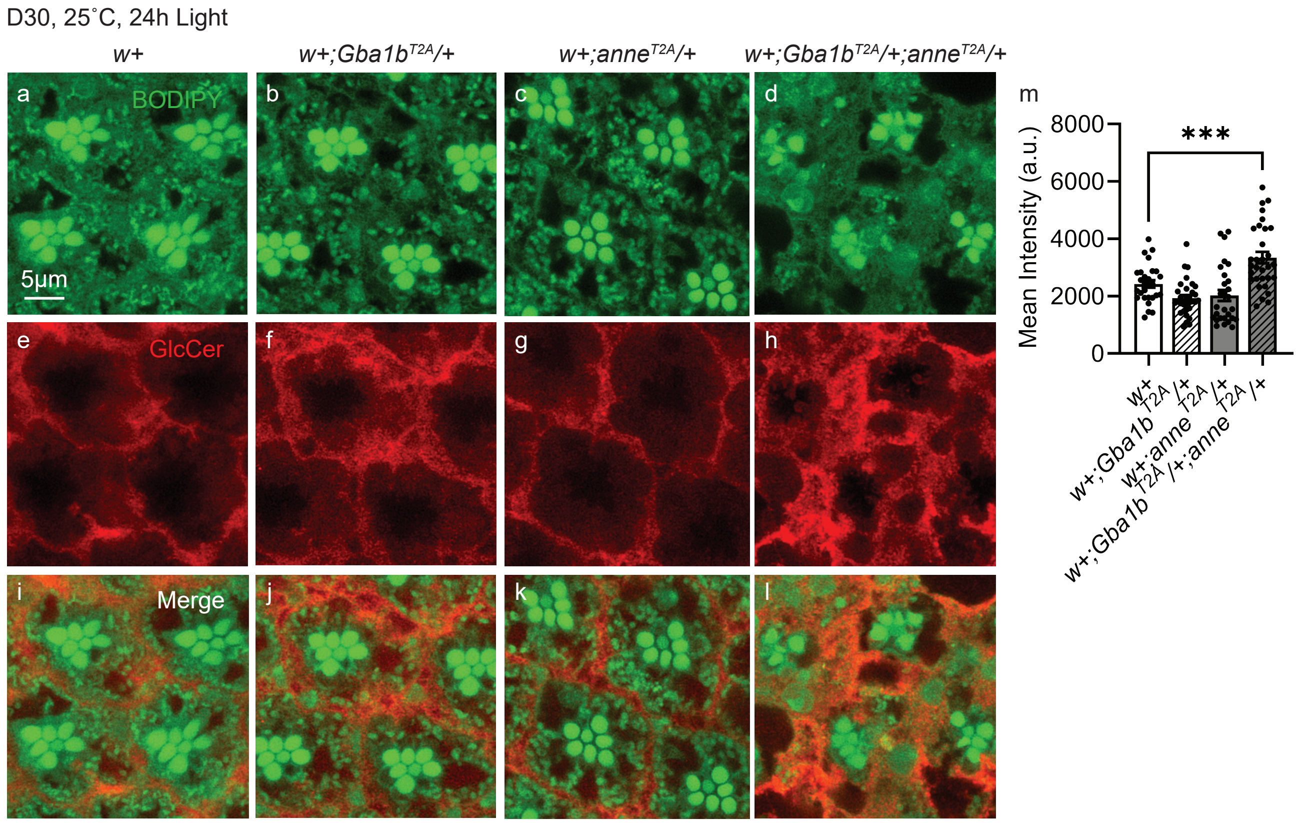

Fig S9 The neurodegenerative phenotypes in *Gba1b*^{T2A}/+;*anne*^{T2A}/+ flies are activity-dependent

A *w+;Gba1b*^{T2A}/+;*anne*^{T2A}/+ flies show no age-dependent ERG defects when not exposed to light

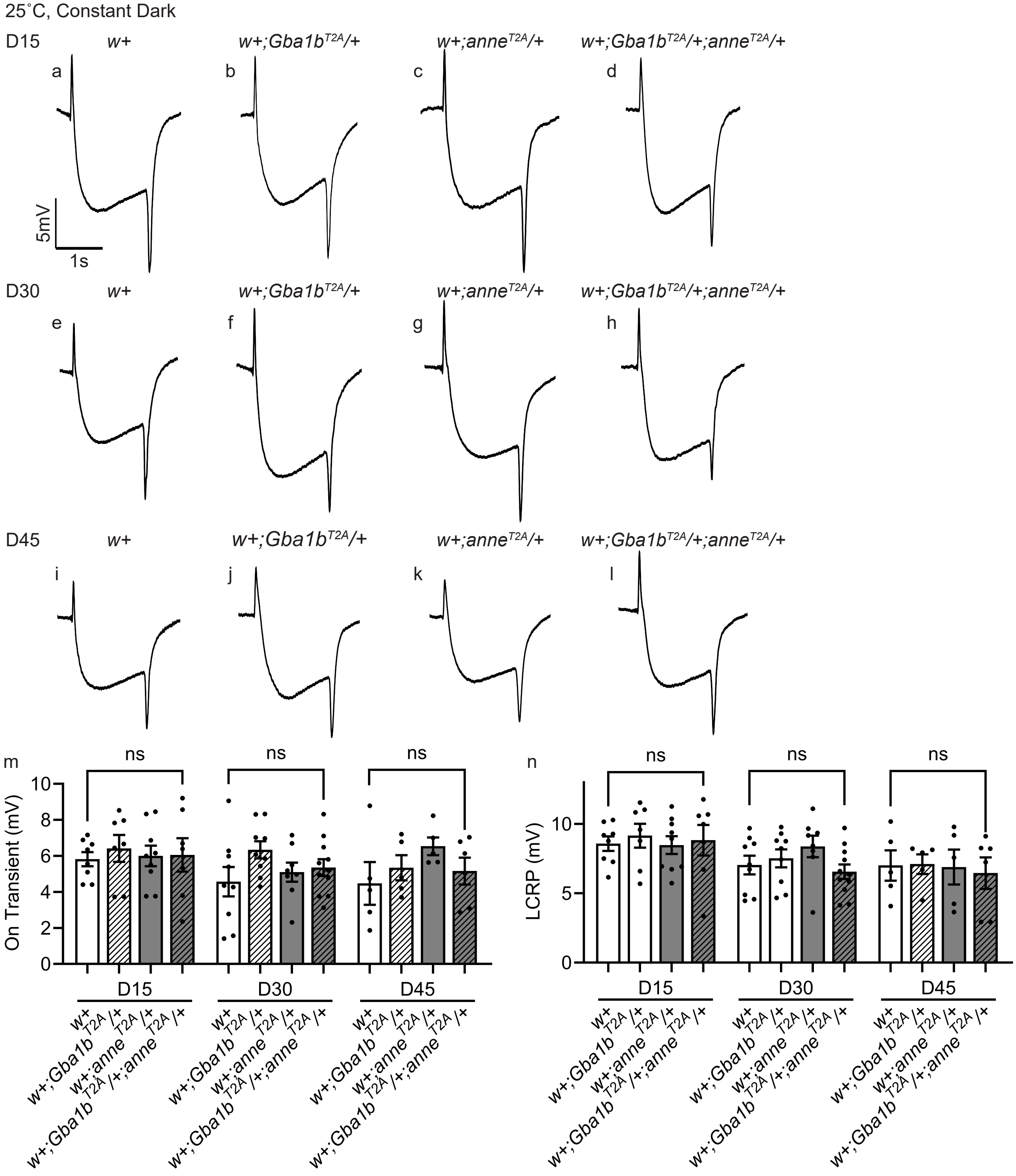

Table S3. Carriers of *GBA1* and *ATP13A2* variants

Individual	*GBA1* variants	*ATP13A2* variants	Sex	Age	Diagnosis	Genotype method
1	p.N409S	p.Y1020Tfs*3	Male	76.868	PD	WGS
2	p.N409S	p.Y1020Tfs*3	Male	68.8	PD	WGS
3	p.T408M/ p.E365K	p.Q635*	Male	73	PD	WGS/CES
4	p.N409S	p.Y1020Tfs*3	Male	76.868	PD	WGS
5	rs3115534-G	c.477+2T>G	Male	57	PD	Array/CES

* participant 4 is a homozygous carrier of *GBA1* p.N409S.

We only included carriers of *ATP13A2* variants that were classified as “pathogenic” or “likely pathogenic” by the ACMG guideline. WGS: whole genome sequencing; CES: clinical exome sequencing.

Table S4. Information of *ATP13A2* variants[1]

Variant	Functional Consequence	Germline Classification	GP2 PD carrier overall	GP2 PD frequency	GP2 GBA1-PD carrier	GP2 GBA1-PD frequency	UKB carrier overall	UKB frequency	UKB GBA1-control carrier overall	UKB GBA1-control frequency	gnomAD global frequency	CADD Phred score
p.Y1020Tfs*3	Frameshift	Pathogenic	6/15881	1.89E-04	2/1292	7.74E-04	28/490530	2.85E-05	1/25869	1.93E-05	6.63E-05	32
p.Q635X	Stop gain	Pathogenic	1/15881	3.15E-05	1/1292	7.74E-04	39/490547	3.98E-05	0/25869	0	2.85E-05	42
c.477+2T>G	Splice donor	Pathogenic	2/15881	6.30E-05	2/1292	1.55E-03	86/490530	8.77E-05	0/25869	0	7.99E-05	18.3
p.R924H	Missense	Likely Pathogenic	1/15881	3.15E-05	0/1292	0	211/490546	2.15E-04	10/25869	1.93E-04	1.44E-04	27.2
p.D513N	Missense	Likely Pathogenic	0/15881	0.00E+00	0/1292	0	2/490544	2.04E-06	1/25869	1.93E-05	1.86E-06	26.5
p.G1122Vfs*17	Frameshift	Likely Pathogenic	0/15881	0	0/1292	0	1/490518	1.02E-06	1/25869	1.93E-05	6.20E-07	32
p.Gln1140*	Stop gain	Likely Pathogenic	0/15881	0	0/1292	0	1/490527	1.02E-06	1/25869	1.93E-05	6.34E-07	47
c.2412+1G>T	Splice donor	Likely Pathogenic	0/15881	0	0/1292	0	3/490531	3.06E-06	1/25869	1.93E-05	6.80E-07	33
p.R449*	Stop gain	Pathogenic	0/15881	0	0/1292	0	11/490546	1.12E-05	1/25869	1.93E-05	9.29E-06	33
p.T402M	Missense	Pathogenic	0/15881	0	0/1292	0	25/490530	2.55E-05	1/25869	1.93E-05	2.60E-05	25.5

1 Due to the very low frequency of the implicated variants it is not possible to confidently estimate an odds ratio.

Supplementary References

1. Siju KP, De Backer JF, Grunwald Kadow IC. Dopamine modulation of sensory processing and adaptive behavior in flies. Cell Tissue Res 2021 3831 [Internet]. 2021 Jan 30 [cited 2025 Dec 5];383(1):207–25. Available from: https://link.springer.com/article/10.1007/s00441-020-03371-x
