# Supplementary material for: Two lysosomal genes ATP13A2 and GBA1 interact to drive neurodegeneration
Source: Mol Neurodegener. 2026 Jan 30;21:18. doi: 10.1186/s13024-025-00923-z (PMC13001375; doi:10.1186/s13024-025-00923-z)
Supplement: Supplementary file 5 — Supplementary Material 5 [file 13024_2025_923_MOESM5_ESM.pdf]

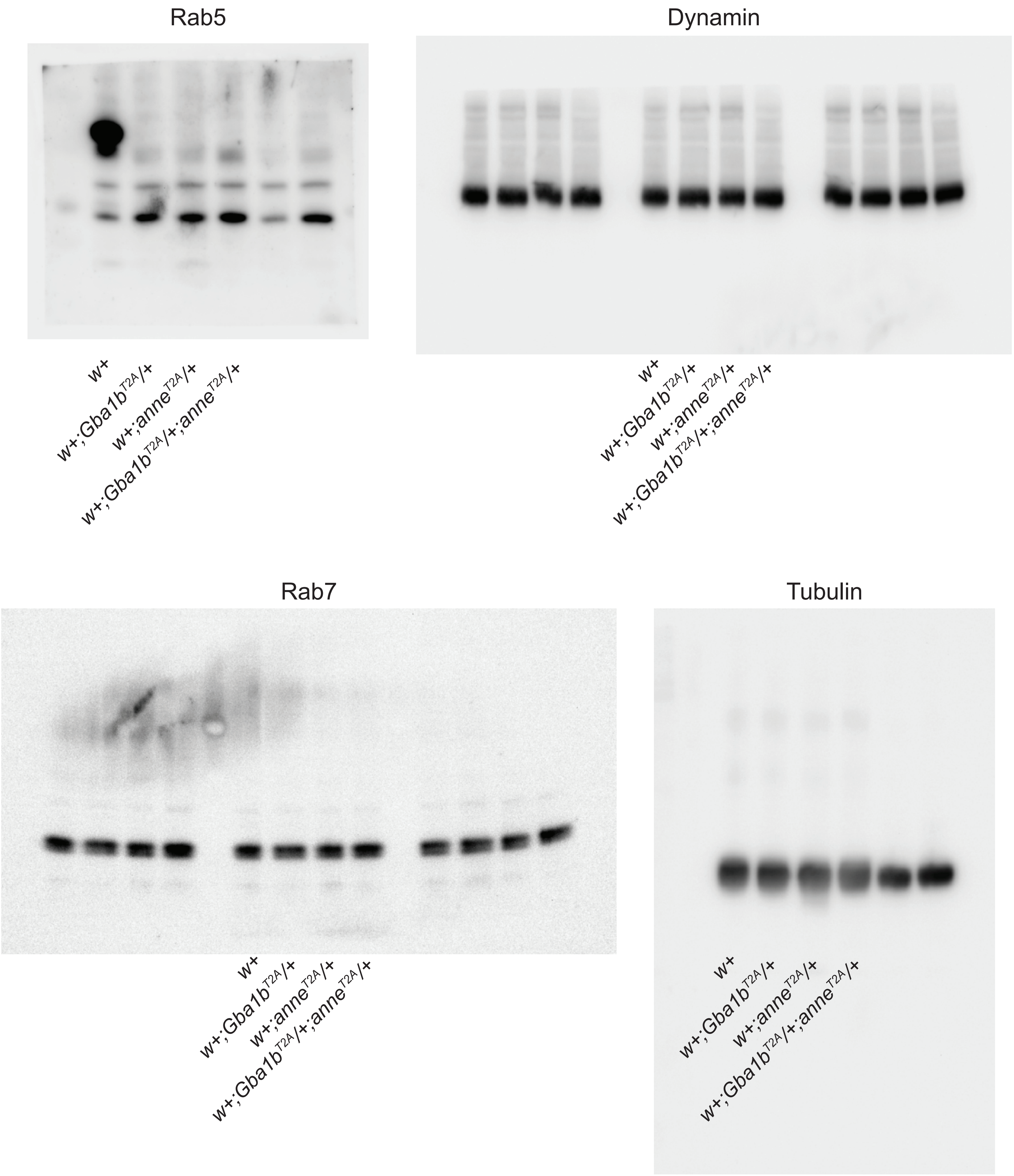

Rab5
w+
w+;Gba1bT2A/+
w+;anneT2A/+
w+;Gba1bT2A/+;anneT2A/+
Dynamin
w+
w+;Gba1bT2A/+
w+;anneT2A/+
w+;Gba1bT2A/+;anneT2A/+
Rab7
w+
w+;Gba1bT2A/+
w+;anneT2A/+
w+;Gba1bT2A/+;anneT2A/+
Tubulin
w+
w+;Gba1bT2A/+
w+;anneT2A/+
w+;Gba1bT2A/+;anneT2A/+

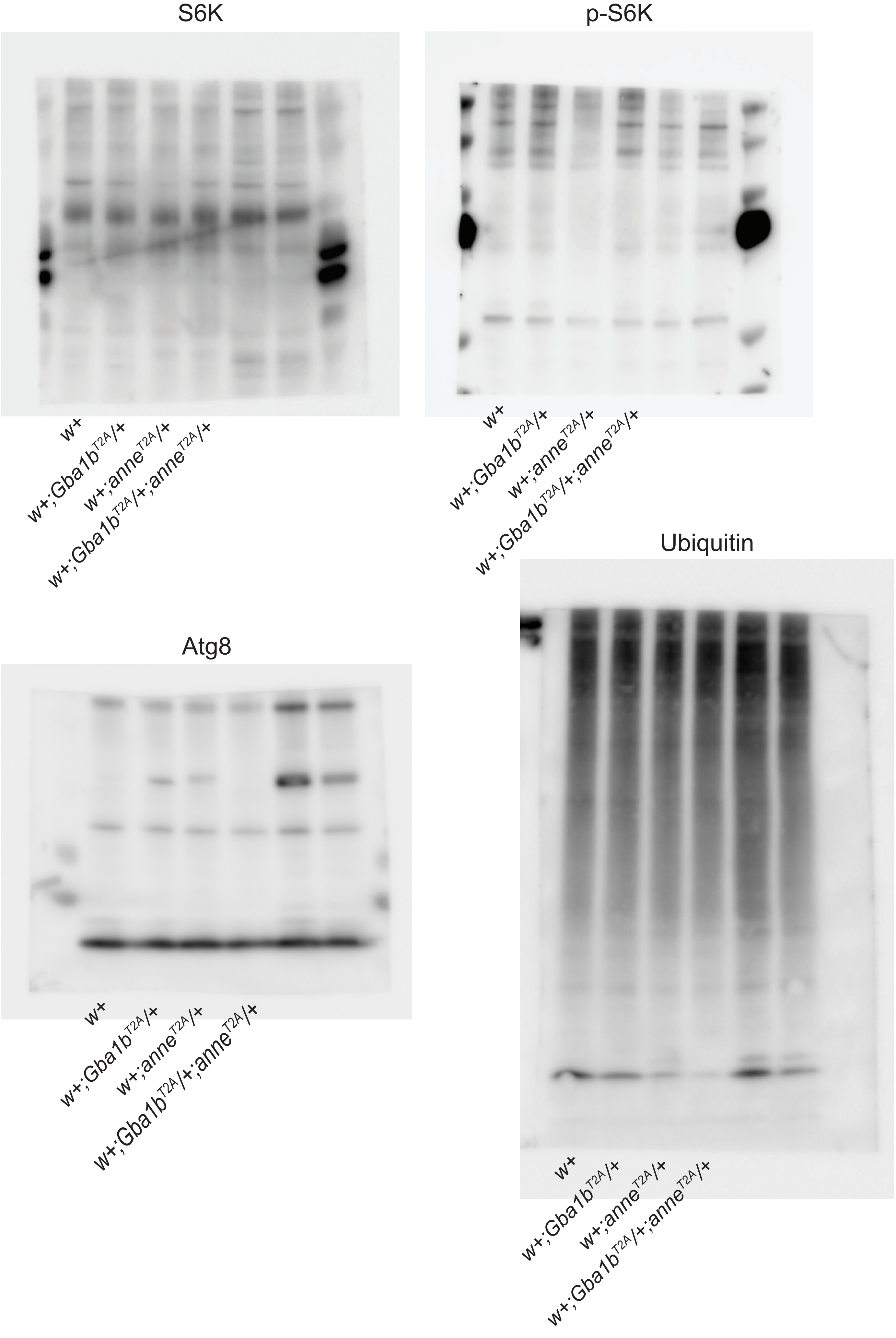
S6K
w+
w+;Gba1b^{T2A}/+
w+;anne^{T2A}/+
w+;Gba1b^{T2A}/+;anne^{T2A}/+
p-S6K
w+
w+;Gba1b^{T2A}/+
w+;anne^{T2A}/+
w+;Gba1b^{T2A}/+;anne^{T2A}/+
Atg8
w+
w+;Gba1b^{T2A}/+
w+;anne^{T2A}/+
w+;Gba1b^{T2A}/+;anne^{T2A}/+
Ubiquitin
w+
w+;Gba1b^{T2A}/+
w+;anne^{T2A}/+
w+;Gba1b^{T2A}/+;anne^{T2A}/+
